# Twice‐Functionalized Montmorillonite Nanosheets for Polymer‐Derived MMT‐SiOC Nanocomposites: Phase Formation and Porosity

**DOI:** 10.1002/smll.202408218

**Published:** 2025-04-07

**Authors:** Advaith V. Rau, Kathy Lu

**Affiliations:** ^1^ Department of Materials Science and Engineering Virginia Polytechnic Institute and State University Blacksburg Virginia 24061 USA; ^2^ Department of Mechanical and Materials Engineering University of Alabama‐Birmingham Birmingham AL 35294 USA

**Keywords:** ceramic nanocomposites, montmorillonite, porosity, silicon carbide, silicon oxycarbide

## Abstract

In this study, montmorillonite (MMT) nanosheets are purified and exfoliated from a crude clay source and further twice‐functionalized with cetritrimethylammonium bromide and [3‐(2‐aminoethylamino)propyl]trimethoxysliane (AEAPTMS) to promote dispersion in the preceramic polymer. Phase profiles and compositions of MMT nanoflakes and MMT‐silicon oxycarbide (SiOC) are characterized with X‐ray diffraction, infrared spectroscopy, and thermogravimetric analysis. The microstructures are examined by scanning and transmission electron microscopy. MMT nanoflakes are randomly dispersed in the SiOC matrix with α‐quartz forming at the MMT‐SiOC interface. Pyrolysis to 1400 °C induced the formation of SiC nanowhiskers that are observed up to 20 µm in length and 200 nm in diameter. After selective etching of SiO_2_ domains with HF, pore sizes and specific surface areas of MMT‐SiOC are analyzed with nitrogen adsorption. The study provided a new fundamental understanding of MMT‐SiOC interactions at different pyrolysis temperatures and also led to composites with specific surface areas reaching 120 m^2^ g^−1^ up to 1200 °C pyrolysis, and between 340 and 772 m^2^ g^−1^ at 1400 °C pyrolysis and pore size distributions between 2 and 5 nm. The methodology and results presented improve the understanding and viability of 2D nanomaterial‐reinforced ceramic composites and MMT as a precursor for nanostructured SiC.

## Graphical abstract

Novel MMT‐SiOC ceramic nanocomposites are prepared. At 900°C and 1200°C, growth and refinement of a crystalline α‐SiO_2_ phase on the MMT nanoflakes led to 2 to 5 nm pores upon etching. The size of the SiO_2_ domains appeared to be diffusion‐limited by turbostratic C phases in the SiOC matrix. Pyrolysis to 1400 °C led to β‐SiC nanowhisker formation and hexagonal faceting.

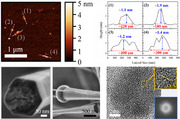

## 1 Introduction

Polymer‐derived ceramics (PDCs) offer a novel yet facile method to fabricate complex ceramics with tailored microstructures and properties by choice of polymer precursor chemistry and filler materials.^[^ ^1^, ^2^, ^3^, ^4^ ^]^ The tunable composition control and the processability ease of the polymer precursors make PDCs an exciting material for synthesizing and fabricating novel materials with complex geometries generally unobtainable or difficult through conventional ceramic processing techniques. In particular, the silicon oxycarbide system (SiO_x_C_y_) has attracted interest for its high thermal stability and low thermal conductivity,^[^ ^5^ ^]^ excellent corrosion resistance,^[^ ^6^ ^]^ and attractive thermomechanical properties.^[^ ^7^ ^]^ Beyond modifications of the polymer precursor chemistry, additional or secondary functionalities have been imbued through the incorporation of either active or passive fillers which can be incorporated into the pre‐ceramic matrix. In particular, some additions are aimed at minimizing volume shrinkage and pore formation common in PDC systems. Both active fillers (e.g., Ti, TiSi_2_, Hf) and passive fillers (e.g., SiC, BN, ZrO_2_, Al_2_O_3_) have been utilized and shown to reduce volume shrinkage,^[^ ^8^, ^9^, ^10^, ^11^ ^]^ impart magnetic,^[^ ^12^, ^13^ ^]^ electric,^[^ ^5^, ^14^ ^]^ or catalytic^[^ ^15^ ^]^ behavior, and improve high‐temperature oxidation behavior.^[^ ^16^, ^17^ ^]^ However, these fillers can potentially be expensive or yield the formation of undesirable secondary phases in the material. Analogous to applications in polymer composites, clay minerals can be a viable, inexpensive, and attractive option to improve the physical properties of SiOC ceramics, but as of yet have not been extensively studied.

Montmorillonite (MMT) is a naturally occurring 2:1 layered aluminosilicate mineral, with stacked sheets of ≈1 nm thickness comprising of an alumina octahedral sheet between two silica tetrahedral sheets.^[^ ^18^, ^19^, ^20^, ^21^ ^]^ MMT composition is generalized as M_0.33_(Al,Mg,Fe)_2_(Si_4_O_10_)(OH)_2_·*n*H_2_O, in which M represents intercalating cations (e.g., Na^+^, Ca^+^), and some inherent water intercalation is present. Due to isomorphous substitutions of Mg^2+^, Al^3+^, and Fe^2+^ among the octahedral and tetrahedral sheets, the MMT layers are inherently negatively‐charged and are balanced by low valence cations.^[^ ^18^, ^20^, ^22^ ^]^ As a result, MMT exhibits a well‐known swelling capability in the presence of water through the hydration of the interlayer cations.^[^ ^19^, ^20^, ^21^, ^23^ ^]^ Furthermore, MMT sheets are held together only by weak electrostatic and van der Waals forces, so MMT is capable of exfoliating into distinct nanometer‐thick sheets.^[^ ^20^, ^21^ ^]^

Obtaining exfoliated microstructures in MMT‐polymer composites remains an issue due to the inherent stacking tendencies of MMT layers to form intercalated, tactoid, or aggregated microstructures.^[^ ^24^ ^]^ Serving as crack or defect initiation sites, agglomerated MMT has been observed to reduce composite mechanical properties. Twice‐functionalized MMT of commercially available hydrophobic bulk MMT has been reported to improve dispersion, intercalation, and exfoliation in polymer matrices.^[^ ^25^, ^26^, ^27^ ^]^ By utilizing long alkyl chain ammonium surfactants to preemptively increase the MMT interlayer spacing, silane molecules are less hindered sterically and chemically from intercalating and grafting to the inner regions of MMT sheets.^[^ ^27^ ^]^ Overcoming the intrinsic layering tendencies of MMT is tantamount to achieving a homogenous exfoliated microstructure in the preceramic green body and pyrolyzed ceramic nanocomposite. MMT nanosheets were reported to exfoliate into single‐to‐few layers by freezing, thawing, and sonicating an aqueous suspension of bulk MMT, which could be obtained after lyophilization.^[^ ^19^, ^20^, ^21^ ^]^ The prominent volumetric expansion associated with water freezing provided adequate force to separate and exfoliate MMT nanosheets after only a few cycles and in conjunction with sonication. By initially exfoliating MMT layers, the nanosheets can be functionalized with colloidal and conventional nanomaterial processing techniques in order to retain the exfoliated microstructure in the preceramic green body and ceramic nanocomposite. Bernando et al. reported preliminary studies of MMT as an additive in a polymer‐derived SiO_x_C_y_ system from a commercial polysiloxane.^[^ ^28^ ^]^ Compared to the native glass, SiOC‐MMT ceramics with 30 and 40 wt.% commercially‐available hydrophobic MMT showed improved hardness and fracture toughness. However, several issues regarding structural integrity of, pore evolution, and crack formation in the pyrolyzed ceramic limited the study to the aforementioned MMT concentrations, and nanostructured MMT was not utilized as the long alkyl chain ammonium surfactant was removed prior to composite fabrication.

In this study, novel polymer‐derived MMT‐SiOC nanocomposites were fabricated with exfoliated and twice‐functionalized MMT nanosheets obtained by freeze/thaw/sonication‐assisted exfoliation of a naturally occurring MMT clay. MMT nanosheets were exfoliated and modified with cetyltrimethylammonium bromide (CTAB) and [3‐(2‐aminoethylamino)propyl]trimethoxysliane (AEAPTMS) to improve dispersion and exfoliation in the preceramic polymer. MMT content and pyrolytic processing temperature were varied to evaluate the effect of MMT on phase evolution and selectively etched porosity in MMT‐SiOC nanocomposites.

## 2 Results and Discussion

### 2.1 MMT Nanosheet Exfoliation and Functionalization

**Figure 1:**
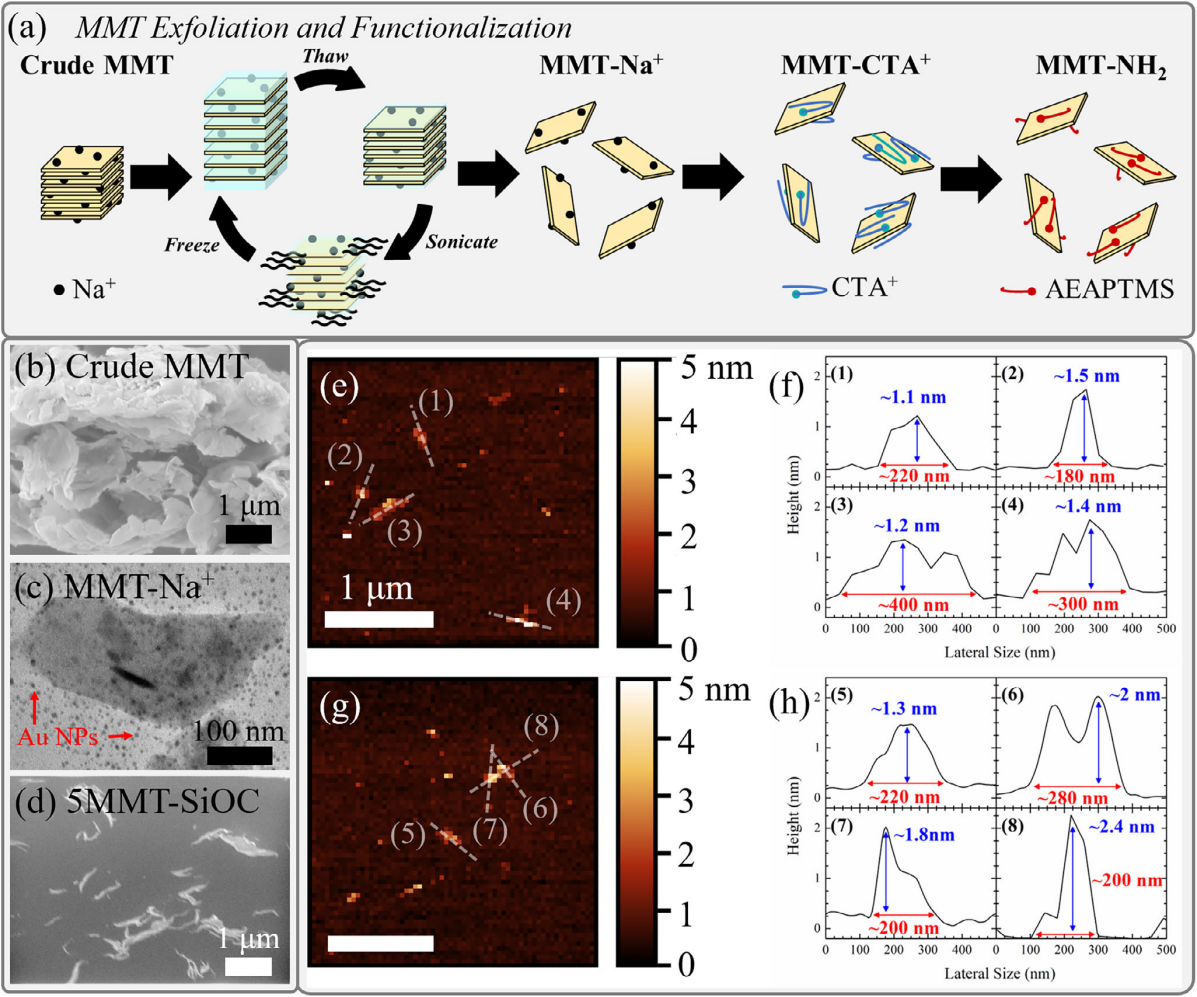
a) Schematic of organophilic MMT nanoflakes synthesized with exfoliation via freeze/thaw/sonication and twice‐functionalization with CTAB and AEAPTMS, b) morphology of crude MMT powders before exfoliation scale bar: 1 µm), c) TEM micrograph of MMT‐Na^+^ nanoflakes after exfoliation (scale bar: 100 nm), and d) cross‐section (prepared by FIB lift‐out) SEM micrograph of 5MMT‐SiOC composite pyrolysis showing randomly exfoliated microstructure of MMT domains in the SiOC matrix (scale bar: 1 µm); e,g) AFM images of MMT‐Na^+^ nanoflakes with false coloring for clarity (scale bars: 1 µm); f,h) selected line profiles as indicated by (1)‐(8) in (e)/(g) displaying typical lateral size (red) and thickness (blue) measurements of MMT‐Na^+^ nanoflakes.

MMT nanosheets were required to colloidally disperse/exfoliate in preceramic PSO in order to obtain exfoliated MMT‐SiOC ceramic microstructures. Not only did this involve overcoming the inherent material incoherency between hydrophilic MMT and organophilic PSO, but also required a novel fabrication route to obtain an appropriate yield (order of grams) of exfoliated MMT nanosheets for bulk sample preparation. **Figure** **1**a depicts the synthesis of exfoliated, hydrophobic MMT through freeze/thaw/sonication (F/T/S)‐mediated exfoliation and subsequent functionalization with CTAB and AEAPTMS. The F/T/S method leveraged the swellability of MMT due to interlayer Na^+^ cations and the volumetric expansion of water upon freezing to exfoliate and isolate MMT nanosheets with yields comparable to those reported previously.^[^ ^19^, ^20^, ^29^ ^]^ While chemically identical, crude MMT and MMT‐Na^+^ were morphologically distinct as the latter referred to single‐to‐few layer thick nanosheets obtained after F/T/S exfoliation of the multilayered/bulk structure in the former. Colloidally stable in water, the hydrophilic exfoliated MMT‐Na^+^ nanoflakes were further processed to yield organophilic, covalently‐bonded functionality to the MMT nanoflakes. CTAB was employed as an intermediate surfactant rather than an intercalating agent to prevent nanosheet agglomeration after exfoliation but prior to silane functionalization. CTAB was not chosen as the terminal functional group as volatilization of the long alkyl chain could induce porosity in pyrolyzed MMT‐SiOC ceramics. However, directly functionalizing MMT with silanes produced incomplete surface covering, agglomeration, and silane bridging in part owing to the basicity and high surface energy of the MMT nanosheets.^[^ ^30^, ^31^, ^32^, ^33^, ^34^ ^]^ In essence, CTAB served as a capping agent akin to traditional nanoparticle synthesis to lower the surface energy to promote dispersion in organic media that facilitate monolayer silane grafting.^[^ ^25^, ^27^ ^]^

Crude MMT powders exhibited highly agglomerated yet sheetlike morphology as shown in Figure 1b. MMT nanoflakes after exfoliation (Figure 1c) showed electron transparency in TEM, indicating single‐to‐few sheet thickness. Nanoflakes after exfoliation were ≈300 nm in length and 200 nm in width. Au NPs, deposited on the TEM grid prior to loading MMT nanoflakes to mitigate charging and electron beam damage, were observable through the nanosheet. MMT nanosheets obtained an exfoliated microstructure in the eventual MMT‐SiOC ceramic (Figure 1d) as shown in the FIB cross‐section lamella of 5MMT‐900. While there was some stacking due to the high weight content, MMT nanosheets were randomly oriented and distributed in the SiOC matrix. MMT nanoflake size and thickness were evaluated with AFM mapping (Figure 1e,g) which revealed generally monolayer (thickness ≈1 nm) and with lateral sizes between 200 and 400 nm obtained from line profiles (Figure 1f,h). MMT‐Na^+^ nanosheets were slightly prone to restacking into bilayers as shown by sheet thicknesses of ≈2 nm in line profiles 6, 7, and 8 (Figure 1g,h). Overall, MMT‐Na^+^ nanoflakes were predominantly monolayer with aspect ratios between 200 and 300. Generally higher aspect ratio nanosheets are desirable to fully leverage the high specific surface areas for interfacial bonding between the matrix and the nanosheets. While providing a greater reinforcement efficiency, larger nanosheets have the potential for agglomeration and could serve as discontinuous inclusions, causing cracks or pores. While smaller nanosheet size may lead to better dispersion, a higher loading content would be needed to obtain similar properties of composites with larger nanosheets.

**Scheme 1:**
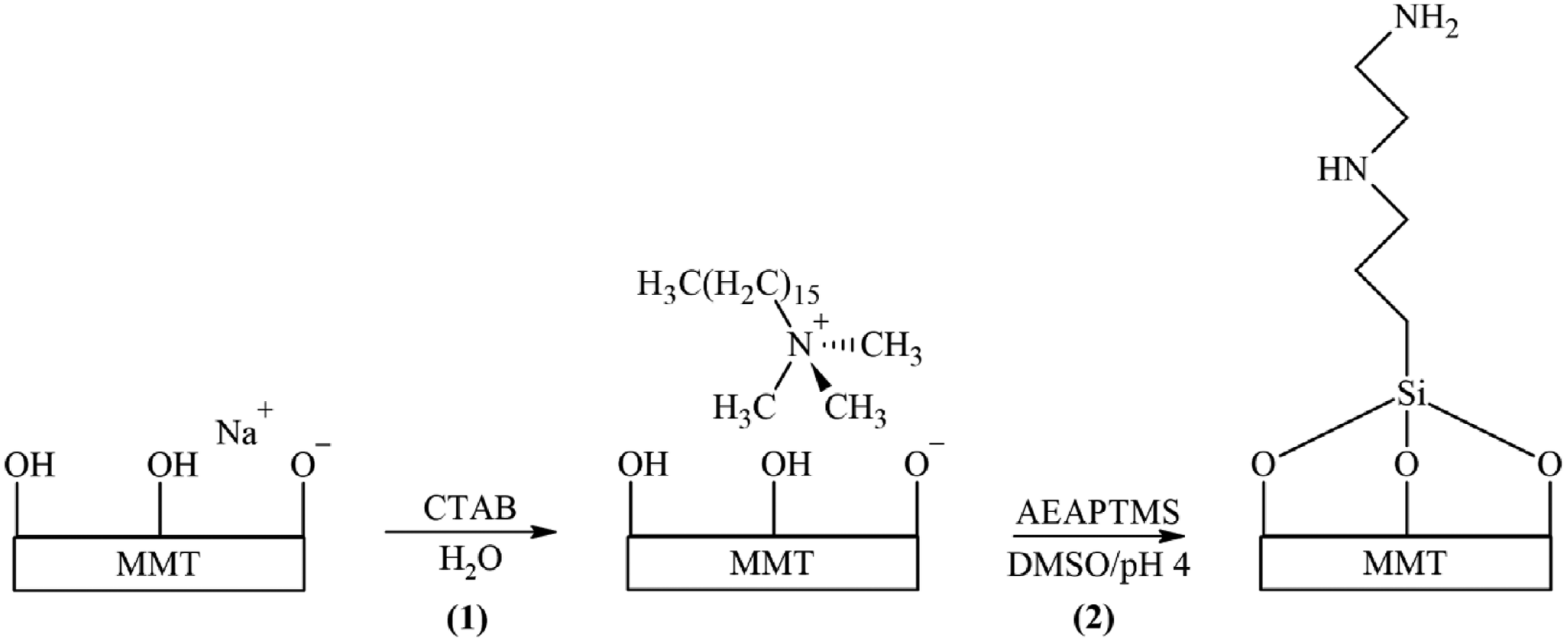
Twice‐functionalization of MMT nanosheets. (1) Cationic exchange of Na^+^ with CTA^+^, (2) Acid‐catalyzed silane (AEAPTMS) condensation.

In order to generate covalently‐bonded silane surface groups, twice‐functionalization was necessary with commercial MMT clays as the direct synthesis route yielded agglomerated MMT layers, interlayer bridging via silanes, or inadequate surface functionalization.^[^ ^31^, ^35^ ^]^ While obtaining fully exfoliated polymer‐clay microstructures remains difficult with twice‐functionalized commercial MMT clays, the F/T/S exfoliation route was chosen to isolate and colloidally process nanosheets to achieve proper dispersion in the preceramic polymer. Initial functionalization with CTAB occurred through the cationic exchange of CTA^+^ with Na^+^ at the MMT nanosheet surface (**Scheme** **1** – Reaction 1). While electrostatically coordinated, CTA^+^ ligands imbued organic functionality that mitigated nanosheet agglomeration before colloidal suspension in the preceramic polymer. Silane functionalization of MMT‐CTA^+^ proceeded by removal of CTA^+^ ligands in an acidic medium and subsequent grafting of AEAPTMS (Scheme 1 – Reaction 2). Acid‐catalyzed AEAPTMS condensation was performed to imbue a near‐monolayer organophilic coating without silane condensate by‐products.^[^ ^36^ ^]^ As the organic surface functionalities were expected to evolve during PDC pyrolysis, excess silane molecules could contribute to additional mass loss and pore formation.

**Figure 2:**
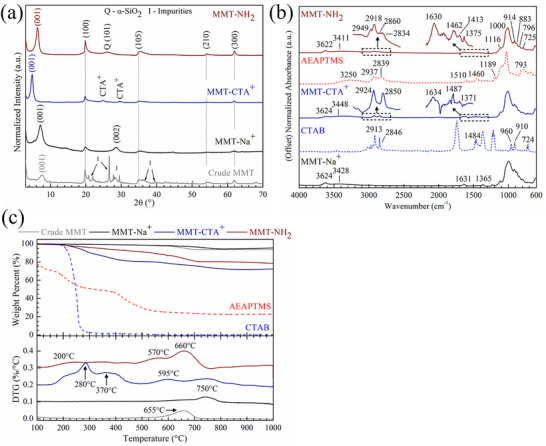
Structural and chemical characterization of exfoliated and functionalized MMT nanoflakes. a) XRD spectra of crude MMT (gray), MMT‐Na^+^ (black), MMT‐CTA^+^ (blue), and MMT‐NH_2_ (red) nanoflakes, b) FTIR spectra of MMT‐Na^+^ (black), MMT‐CTA^+^ (blue), and MMT‐NH_2_ (red) nanoflakes, c) TG/DTG profiles of crude MMT (gray), MMT‐Na^+^ (black), MMT‐CTA^+^ (blue), and MMT‐NH_2_ (red). Reference spectra of CTAB (light blue) and AEAPTMS (light red) are provided for comparison in (b)/(c).

**Figure** **2**a depicts the phase profiles of MMT exfoliation and functionalization products from the crude clay source to twice‐functionalized MMT. The evolution of exfoliated MMT nanoflakes that are single‐to‐few layers thick was indicated by the emergence of the (002) basal spacing peak at 7° and subsequent peak shift to smaller scattering angles. Retention of the exfoliated structure throughout the functionalization steps was imperative to preventing agglomeration and layering of the MMT nanoflakes in the preceramic green body. Crude MMT inherently exhibited a bulk, aggregated structure with naturally occurring impurities (e.g., quartz, carbonates, sediments, etc.). After purification and exfoliation of the crude clay, the exfoliated MMT‐Na^+^ nanosheets were characterized by the emergence and sharpening of the (002) diffraction peak at 7° and a general decrease in higher order (00n) peaks. A (002) basal spacing peak at 29° was observed in MMT‐Na^+^ that disappears upon further functionalization, and was attributed to inherent stacking tendencies after lyophilization due to van der Waals forces. Peaks characteristic to the MMT nanosheet (e.g., tetrahedral/octahedral layers) were identified at 20°, 35°, 54°, and 62° as (100), (105), (210), and (300) planes, respectively.

After CTAB functionalization, the (002) basal spacing peak in MMT‐CTA^+^ significantly increases in intensity and shifts from 7° to 4.9°, correlating to an increase in d‐spacing from 12.6 to 18.0 Å, compared to MMT‐Na^+^. Characteristic MMT diffraction peaks for (100), (105), (210), and (300) were retained at 20°, 35°, 54°, and 62°, respectively. Noting that the (002) basal spacing diffraction peak disappeared completely, the MMT nanosheets were determined to be predominantly monolayer and did not agglomerate. The basal spacing increase was induced by the successful grafting of CTAB to the MMT‐Na^+^, as the hydrophobic tail provided a barrier against agglomeration. The two new diffraction peaks observed at 24.5° and 29° (denoted as CTA^+^ in Figure 2a) were attributed to some coherent ordering of the CTA^+^ ligands on the MMT nanoflake substrate when dried. Previous reports indicated that ordered states of intercalated alkyl chains have been observed due to inherent alkyl chain length, increasing chain interactions, and packing density of the alkyl ligands.^[^ ^18^ ^]^ The corresponding d‐spacings for the aforementioned CTA^+^ diffraction peaks were 3.6 Å and 3.1 Å. While the total d‐spacing contribution from CTA^+^ (6.7 Å) was slightly larger than the change in (002) basal spacing (5.4 Å), this discrepancy was attributed to the loosely crystalline packing of CTA^+^ ligands. Overall, the sole presence and refinement of the (002) diffraction and unique CTA^+^ peaks indicated that the exfoliated morphology was retained after CTAB functionalization and CTAB was properly grafted to the nanosheet surface.

Upon functionalization with AEAPTMS, the intensity of the (002) peak significantly increased with the absence of the (004) peak at 29°, indicating that the MMT‐NH_2_ nanoflakes were thoroughly exfoliated and predominantly single layer. In addition, the (002) peak shift from MMT‐Na^+^ to twice‐functionalized MMT‐NH_2_ of 7° to 5.5° corresponded to an increase of basal spacing from 12.6 Å to 16.0Å, respectively. Intrinsic MMT nanoflake diffractions of (100), (105), (210), and (300) planes were similarly observed at 20°, 35°, 54°, and 62°, respectively. Notably, a new diffraction peak at 26° after AEAPTMS functionalization was attributed to the silane monolayer formed on the MMT nanosheet surface. As silane functionalization occurs through covalent linkage with the exposed Si─O groups of the MMT nanosheet, an effective siliceous layer forms through a bridging O atom between Si atoms of the MMT nanosheet and the silane. The d‐spacing of the (101) SiO_2_ peak was 3.4 Å, which directly correlates to the difference in (002) basal spacing between MMT‐Na^+^ and MMT‐NH_2_. While the (002) basal spacing decreased from d‐spacings of 18.0 Å to 16.0 Å from MMT‐CTA^+^ to MMT‐NH_2_, the absence of higher order (00*n*) diffraction peaks indicated that the MMT monolayers were retained without agglomeration. The decrease in d‐spacing was attributed to the difference in hydrophobic tail length between CTAB (18 C atoms) and AEAPTMS (5 C/2 N atoms). Not only was the exfoliated structure retained after functionalization, successful grafting of AEAPTMS to the MMT nanosheet surface increased the equilibrium spacing and mitigated nanoflake aggregation or stacking.

Bond formations in MMT‐CTA^+^ and MMT‐NH_2_ were further elucidated with FTIR‐ATR and XPS to observe CTAB or AEAPTMS motifs in the functionalized MMT products. Figure 2b depicts the FTIR spectra of MMT‐Na^+^ (black), MMT‐CTA^+^ (red), and MMT‐NH_2_ (blue) along with the reference spectra for CTAB (dashed blue) and AEAPTMS (dashed red). MMT‐Na^+^, MMT‐CTA^+^, and MMT‐NH_2_ all exhibited identical bands in the 1200–700 cm^−1^ region that are due to stretching vibrations of Si─O(H), Al─O(H), Si─O─Si, and Si─O─Al groups in the MMT nanosheet.^[^ ^37^ ^]^ For these samples, peak identification indicated these bands varied within ± 3 cm^−1^ (within the resolution used in the analysis); for clarity, these vibrations were only noted in the MMT‐NH_2_ spectra but were identical among the three samples. Bands at 1116 and 1000 cm^−1^ originated from Si─O stretching vibrations, and those at 914, 840, and 725 cm^−1^ were attributed to Al─OH─Al, Si─O─Al, and O─(Al^IV^)─OH stretching vibrations in the interior and on the edges of MMT nanosheets, respectively.^[^ ^37^ ^]^ Absorption bands at 3624 cm^−1^ and 3428 cm^−1^ were due to stretching vibrations of O─H groups of structural ─OH in the Al octahedral layer and hydrogen‐bonded/intermolecular H_2_O, respectively. The Al─OH vibration at 3624 cm^−1^ did not appreciably shift after functionalization, which could indicate these edge Al─OH groups in the octahedral layer were not involved in the reactions with CTAB or AEAPTMS. An O─H symmetric bending vibration was also observed at ≈1634 cm^−1^ and similarly attributed to absorbed H_2_O molecules. The intensity of the prior bands decreased after CTAB and AEAPTMS functionalization as few H_2_O molecules could adsorb to the newly hydrophobic MMT surface. The band at 1365 cm^−1^ was assigned to O─H bending vibrations of structural ─OH groups.

After CTAB functionalization, new adsorption bands were observed in MMT‐CTA^+^ at 2924, 2850, and 1487 cm^−1^ – the former two vibrations from alkyl C─H stretching and the latter from alkyl C─H bending modes. These alkane vibrations were from CTA^+^ cations electrostatically bonded with the MMT nanosheet surface as the vibration frequencies and intensities matched closely with the prominent alkyl C─H bands of CTAB. Furthermore, the vibration at 3448 cm^−1^ shifted from 3428 cm^−1^ in MMT‐Na^+^ as fewer H_2_O molecules could be adsorbed onto the hydrophobic MMT‐CTA^+^ nanosheet surface. This band indicated that any H_2_O molecules present were generally hydrogen‐bonded to exposed ─OH structural groups on the octahedral sheet edges.

Twice‐functionalized MMT‐NH_2_ clearly showed the lack of C─H vibrations characteristic of CTA^+^ as the cation dissociated during AEAPTMS functionalization. Expanded views of the MMT‐NH_2_ FTIR spectra between 1700–1300 cm^−1^ and 3100–2700 cm^−1^ depicted the low‐intensity bands from grafted AEAPTMS. The bands at 3411 cm^−1^ and 1630 cm^−1^ were attributed to N─H stretching and bending vibrations in the hydrophobic silane tail. The retained vibrations at 1413 cm^−1^ and 1375 cm^−1^ were assigned to structural ─OH bending modes of the MMT nanosheet that were not involved in the functionalization. Alkyl C─H stretching vibrations from the hydrophobic tail were identified at 2949, 2918, 2875, 2856, and 2834 cm^−1^, and bending vibrations at 1468, 1462, and 1452 cm^−1^. Bands in the 1600–1500 cm^−1^ range were attributed to C═O vibrations from background or surface‐adsorbed CO_2_. The N─H and C─H bands observed in MMT‐NH_2_ closely resembled those in native AEAPTMS.

CTAB and AEAPTMS functionalization was further confirmed with TGA to identify mass loss events and signature functional groups present in MMT‐CTA^+^ and MMT‐NH_2_. Figure 2c depicts the thermogravimetric (TG) and derivative (DTG) profiles of crude MMT (gray), MMT‐Na^+^ (black), MMT‐CTA^+^(red), and MMT‐NH_2_ (blue) under Ar from 100 to 1000 °C. Both crude MMT and MMT‐Na^+^ experienced ≈5% mass loss and the primary mass loss event correlated to dehydroxylation of the MMT surface at ≈655 °C and ≈750 °C, respectively. While the decomposition temperature of crude MMT observed agreed with prior studies, the shift in dehydroxylation temperature for exfoliated MMT nanoflakes has not yet been reported. The temperature shift is indicative that more energy is required to convert surface ─OH groups as water after exfoliation. In a bulk/aggregated form, the closer stacking of MMT sheets is more conducive to dehydroxylation as more mass is available for H_2_O evolution and the layered morphology stabilizes the decomposition of aluminosilicate product at lower temperatures. Upon exfoliation, the MMT nanosheets are sufficiently spaced to retard this decomposition event for an additional ≈100 °C, possibly as the surface energy of the surface ─OH bonds increases with the larger exposed surface area.

After both functionalization with CTAB and AEAPTMS, MMT‐CTA^+^ and MMT‐NH_2_ exhibited higher mass losses of 29% and 21%, respectively, compared to MMT‐Na^+^ after heating to 1000 °C. The reference spectra of CTAB (dashed red) and AEAPTMS (dashed blue) in Figure 2a indicated that CTAB fully decomposed by 300 °C while AEAPTMS yielded a 15% mass residue of siliceous products. The corresponding DTG profiles of MMT‐CTA^+^ and MMT‐NH_2_ illustrated additional gradual mass loss events between 200 and 800 °C, which can solely be attributed to grafted organic molecules after each functionalization step. The gradual mass loss observed in organically‐modified MMT has been reported as the decomposition of hydrogen/electrostatically‐bonded organic molecules between 200 and 400 °C and intercalated/covalently‐bonded molecules above 450 °C.^[^ ^31^ ^]^ The DTG profile of MMT‐CTA^+^ in Figure 2b, the primary decomposition event between 200 and 400 °C, was attributed to CTA^+^ cations coordinated weakly to ─OH/‐O^−^ groups along the surfaces and edges of the MMT nanosheets. From 600 to 800 °C, tightly held CTA^+^ cations that formed an initial monolayer on the MMT surface finally evolved along with any remaining ─OH surface groups.

Twice‐functionalized MMT‐NH_2_ depicted a gradual yet distinct mass loss profile compared to MMT‐CTA^+^, which indicated that CTA^+^ cations were sufficiently removed and replaced with silane molecules. Compared to MMT‐CTA^+^, there was significantly less mass loss observed below 400 °C, which could indicate that the majority of silane molecules are covalently bonded to the MMT surface. The predominant mass loss in MMT‐NH_2_ occurred from 500 to 750 °C as the covalent bonds between MMT and silane were cleaved and residual ─OH groups were evolved. Grafted AEAPTMS content on the MMT nanosheets was determined by TGA mass loss between 200 and 600 °C (W_200–600_), as expressed in Equation (1).

(1) $$\mathrm{Silane}\;\mathrm{Content}\;\;\left(\%\right)=\frac{100\times W_{200-600}}{100-W_{200-600}}\;$$

Neglecting dehydroxylation mass loss at 660 °C intrinsic to MMT‐Na^+^ decomposition (5.6%), *W*_200−600_ was determined to be 19.8%, yielding a grafted silane content of 24.7%. The specific surface area (SSA) of MMT was also estimated from the TGA silane mass loss, the surface density of O atoms on the crystalline MMT surface (σ_*O*−*MMT*_), the grafting ratio of MMT surface O atoms to AEAPTMS Si atoms (*N*_*O*−*Si*_), the molar mass of AEAPTMS (µ_*AEAPTMS*_), and Avogadro's number (*N_a_*), as given in Equation (2).

(2) $$\mathrm{SSA}\;=\frac{\mathrm{Silane}\;\mathrm{Content}\left(\%\right)}{100}\;\;\times \frac{N_{a}}{\mu_{\mathrm{AEAPTMS}}}\times \frac{N_{O-\mathrm{Si}}}{\sigma_{O-\mathrm{MMT}}}$$

σ_*O*−*MMT*_ was approximated as ≈ 1.629 × 10^19^ atom m^−2^ from tetrahedrally‐ordered O atoms on the MMT surface and a crystalline Si─O bond distance of 163 pm, and *N*_*O*−*Si*_ as 3, assuming complete grafting of the silane monolayer (Scheme 1 – Reaction 2). The SSA of MMT‐NH_2_ nanoflakes was calculated to be ≈123 m^2^ g^−1^ from Equation (2), which agreed with a reported value for exfoliated MMT similarly processed through F/T/S exfoliation.^[^ ^38^ ^]^ While SSA values are lacking for MMT nanoflakes, bulk MMT SSA values were generally reported between 10 and 50 m^2^ g^−1^,^[^ ^38^, ^39^, ^40^, ^41^ ^]^ which further pointed to the nanostructured/exfoliated morphology of the twice‐functionalized MMT‐NH_2_ nanoflakes.

### 2.2 MMT‐SiOC Pyrolysis and Properties

#### 2.2.1 Phase Evolution and Physical Properties

**Figure 3:**
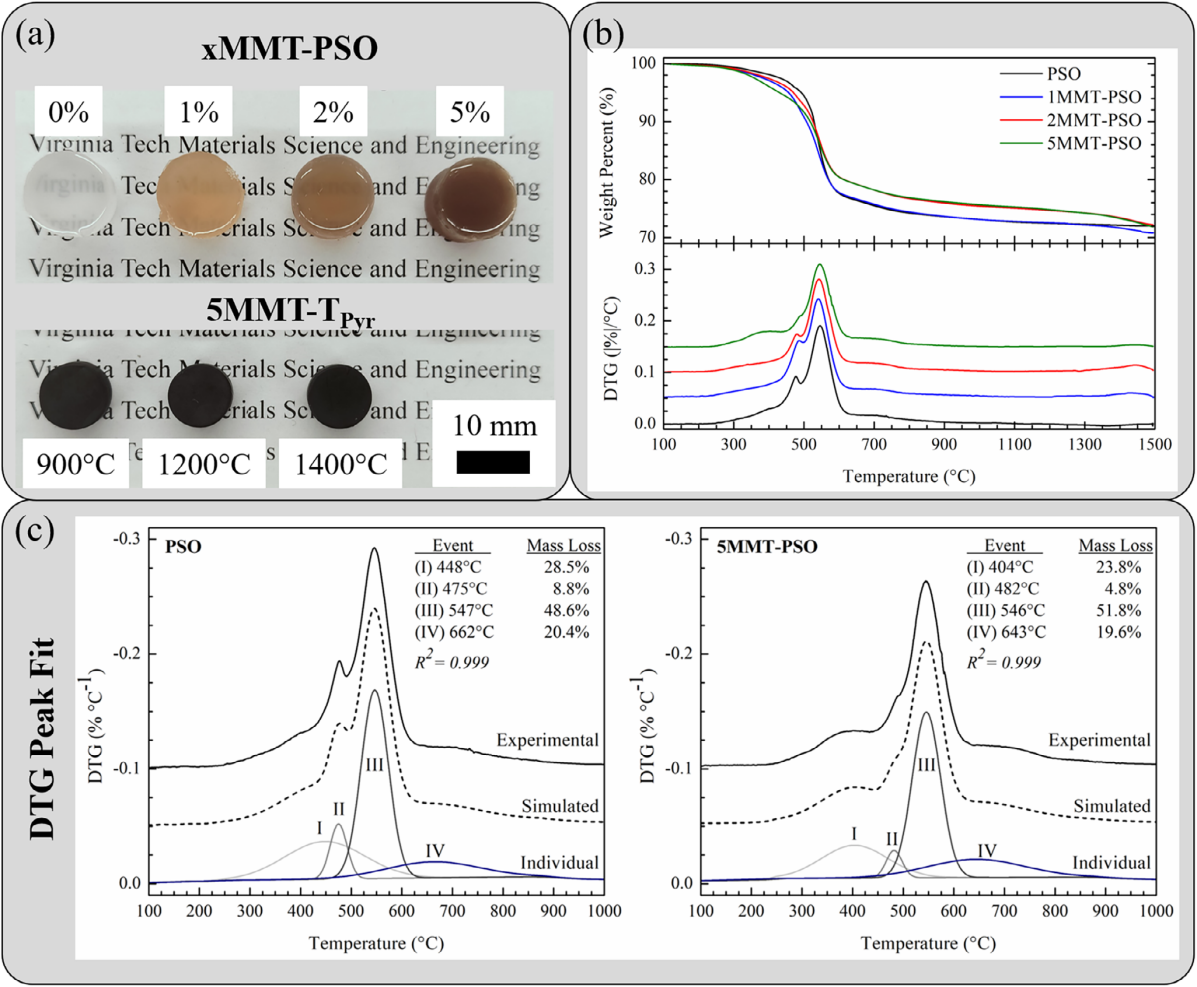
a) Optical image of *x*MMT‐PSO (*x* = 0, 1, 2, 5 wt.%) preceramic green bodies (*top*) and pyrolyzed 5MMT‐900, 5MMT‐1200, and 5MMT‐1400 ceramics (*bottom*); b) TGA (*top*) and DTG (*bottom*) analysis of PSO‐*x*MMT green bodies to 1500 °C in Ar; c) DTG peak fitting analysis of PSO and 5MMT‐PSO between 100 and 1000 °C to evaluate effect of MMT during polymer‐to‐ceramic transformation.

Preceramic *x*MMT‐PSO green bodies (**Figure** **3**a) exhibited colloidal dispersions of functionalized MMT‐NH_2_ nanoflakes, with the yellow color in 1MMT‐PSO darkening to amber and brown in 2MMT‐PSO and 5MMT‐PSO, respectively. Notably, the loss of translucency in 5MMT‐PSO implied that at such a high nanoflake content, MMT nanoflakes may exist as a few sheet‐thick tactoids randomly dispersed in the matrix. Pyrolyzed 5MMT‐900, 5MMT‐1200, and 5MMT‐1400 (Figure 3a) were dense and black from excess free C present in all silicon oxycarbide systems, and were representative of all pyrolyzed MMT‐SiOC samples at the listed temperatures. Pyrolytic decomposition behaviors of *x*MMT‐PSO to *x*MMT‐SiOC were observed with TGA to 1500 °C in Ar (Figure 3b) to determine the effect of MMT on SiOC phase evolution during the polymer‐to‐ceramic transformation (400–800 °C) and up to SiOC carbothermal reduction to SiC domains (>1400 °C). Native PSO yielded a ≈72% residue upon pyrolysis to 1400 °C and no appreciable mass loss was observed up to 1500 °C. 1MMT‐PSO exhibited a similar residue of ≈72% at 1400 °C but displayed additional mass loss (≈1%) up to 1500 °C. 2MMT‐PSO and 5MMT‐PSO yielded slightly higher ceramic residues (≈74%) at 1400 °C with a similar decomposition behavior as 1MMT‐PSO between 1400 and 1500 °C. As not present in PSO, the high temperature decomposition event was not only due to MMT phase separation to cristobalite and mullite but also reactions with the SiOC matrix that would account for gas volatilization. While 1MMT‐PSO and 2MMT‐PSO showed similar DTG profiles to PSO, an additional event at ≈370 °C was revealed in 5MMT‐PSO along with a reduction in mass loss at ≈470 °C. Increasing MMT content lowered the onset of the polymer‐to‐ceramic transition, most prevalently in 5MMT‐PSO, pointing to a reaction between MMT nanosheets and the transforming SiOC matrix.

Pyrolytic decomposition events during the polymer‐to‐ceramic transformation were the combination of multiple individual kinetic processes that cannot be resolved solely with TGA. However, each event may be described by a rate‐limiting step that ultimately drives volatilization reactions observable with TGA. Dedicated kinetic studies with TGA‐ mass spectrometry (MS) for evolved gas characterization would be needed for a more robust analysis of the polymer‐to‐ceramic transition, which was outside the scope of this study. The specific polysiloxane used in this study has not yet been analyzed with TGA‐MS to characterize the evolved gaseous species, but overall similarities with previously studied PDC‐SiOC/SiC systems sufficed for analysis of mass loss events in regards to underlying phase evolution.^[^ ^42^, ^43^, ^44^ ^]^ DTG peak deconvolution for PSO and 5MMT‐PSO (Figure 3c) revealed four decomposition events during the polymer‐to‐ceramic transition. In PSO, events I (448 °C), II (475 °C), III (547 °C), and IV (662 °C) contributed 28.5%, 8.8%, 48.6%, and 20.4% to mass loss during the polymer‐to‐ceramic transformation, respectively. A previous study correlating gas evolution with pyrolytic decomposition events in SiOC derived from 1,3,5,7‐tetramethyl‐1,3,5,7‐tetravinylcyclostetrasiloxane showed event I involved the release of gaseous by‐products from Si─OH and Si─H condensation.^[^ ^42^ ^]^ While heavily overlapping with event I, event II involved the redistribution of Si─H, Si─O, and Si─C bonds and the release of some Si species in the process. Events III and IV were attributed to methane and hydrogen evolution from the cleavage of Si─Me, Si─H, Si─O, and Si─C bonds. DTG peak deconvolution for 5MMT‐PSO revealed that event I (404 °C) occurred earlier and contributed only 23.8% of total mass loss. Events II (482 °C), III (546 °C), and IV (643 °C) proceeded similarly as in PSO but with respective mass loss contributions of 4.8%, 51.8%, and 19.6%. The improved ceramic yield in 5MMT‐PSO originated from decreased mass loss during events I and II – the latter potentially indicating increased retention of Si in MMT‐SiOC ceramics. Early‐stage mass loss (event I) was correlated with higher SiO_2_ content in the resultant SiOC ceramic.^[^ ^42^ ^]^

The phenomenological origin of the observed mass loss profiles was believed to be related to how MMT nanosheets are incorporated into the SiOC matrix. It was not understood whether MMT nanoflakes were embedded in the amorphous SiOC matrix, served as discontinuous inclusions, or segregated into the free carbon phase that is intrinsic in carbon‐rich PDC systems. Discontinuous inclusions can serve as defect or crack initiation sites and represent a common issue in MMT composite fabrication. If the MMT nanosheets were to behave as discontinuous inclusions, the MMT‐SiOC ceramics were theorized to be incredibly brittle as the nanosheets would not be stabilized in the matrix or at high temperatures – the high nanosheet surface energy would cause MMT reinforcements as crack initiation sites to resolve this energy mismatch. Alternatively, if MMT nanosheets were to segregate to the free carbon phase – that is, unincorporated in the amorphous SiOC phase – there would be no significant difference in the TG/DTG profiles of PSO‐*x*MMT green bodies compared to native PSO. In either scenario, MMT would not contribute to or affect SiOC phase formation, so the overall ceramic yield would be modeled as independent decomposition events of PSO‐to‐SiOC and MMT‐NH_2_ to kaolin. However, neither of these scenarios would yield the observed results presented in Figure 3b.

Crystalline MMT nanosheets were postulated to be embedded in the amorphous SiOC matrix through an interfacial zone that resolved the coherency mismatch. During the polymer‐to‐ceramic transformation, radical‐mediated reactions among Si, C, and O atoms form the thermodynamically metastable amorphous SiOC matrix – in addition, reactions with MMT surface terminations either innate or residual from AEAPTMS were postulated to occur in conjunction with AEAPTMS decomposition in MMT‐NH_2_ as shown in Figure 2c. This would have also been necessary to stabilize the high surface energies of the MMT nanosheets as the silane functional groups volatilized. Therefore, it was believed that an interfacial zone between MMT nanosheets and the SiOC matrix evolved to stabilize the embedded nanosheets and resolve the material coherency mismatch. This interfacial region was theorized to transition from ordered Si─O/Si─C bonds near the crystalline MMT nanosheet to short‐range/amorphous ordering of Si─O─C clusters toward the bulk SiOC matrix. SiOC phase formation is driven thermodynamically based on the preceramic polymer chemistry and pyrolytic atmosphere.^[^ ^45^ ^]^ The MMT‐SiOC system was inherently silica‐rich as the nanosheets are comprised of 2 SiO_2_ and 1 Al_2_O_3_ units. To maintain the overall ceramic composition, carbon may have been retained to compensate for the effective nanosized SiO_2_ inclusions. Carbon was thought to be retained in this interfacial region between MMT and SiOC. While the MMT nanosheets were exfoliated, the interfacial zones may be sufficiently large – possible given the high specific surface area of the nanosheets – to coalesce above 1% MMT. As regions coalesce, less carbon may be required to resolve the crystalline/amorphous material boundary between MMT and SiOC compared to more dispersed nanosheets.

**Figure 4:**
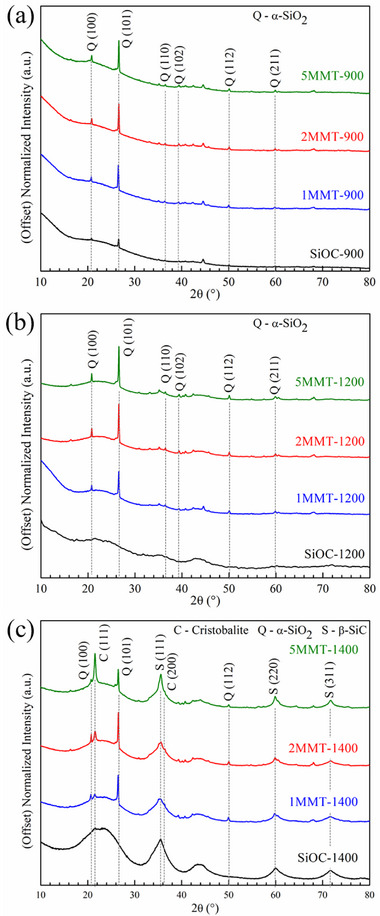
XRD spectra of *x*MMT‐SiOC ceramic nanocomposites after pyrolysis at a) 900 °C, b) 1200 °C, and c) 1400 °C.

**Figure** **4** depicts the phase compositions of *x*MMT‐SiOC at (a) 900 °C, (b) 1200 °C, and (c) 1400 °C as determined by XRD. SiOC‐900 and SiOC‐1200 samples were largely amorphous with the halo from ≈10–40° attributed to amorphous SiOC, SiO_2_, and C domains. A small α‐quartz phase was detected in SiOC‐900 but was attributed to unevolved Si─OH groups from the polymer‐to‐ceramic transition. At 1400 °C (Figure 4c), poorly refined β‐SiC (111), (220), and (311) diffraction peaks at 36°, 60°, and 72°, respectively, emerged from SiOC phase separation as the microstructure developed some nanocrystallinity. However, in the presence of MMT, two diffraction peaks at 20.7° and 26.5° corresponding to the (100) and (101) planes of α‐quartz emerged and were retained in the 1/2/5MMT‐SiOC samples at all temperatures. Overall, the 1/2/5MMT‐SiOC samples were generally amorphous with no other crystalline contributions until 1400 °C. Beyond β‐SiC crystallization inherent to the SiOC matrix, a new cristobalite diffraction peak at 22° developed in the 1/2/5MMT‐1400 samples, and its intensity increased with the MMT content. The MMT‐SiOC samples were summarized as amorphous SiOC ceramics reinforced with nanocrystalline SiO_2_ domains.

While no characteristic nanosheet diffraction peaks were detected, the MMT nanosheets were responsible for cristobalite and α‐quartz crystalline phases present in the MMT‐SiOC nanocomposites. Bulk MMT is known to decompose to metakaolin at ≈650 °C and cristobalite and mullite at ≈1350 °C. The cristobalite phases in 1/2/5MMT‐1400 (Figure 4c) and the corresponding increase in the 22° peak intensity with increasing MMT content indicated that the cristobalite phase was MMT‐derived rather than from the SiOC matrix. No prominent mullite peaks were observed or expected due to the low Al_2_O_3_ content in the MMT‐SiOC system, but should be present in very low concentrations to satisfy the stoichiometric requirements of cristobalite formation from MMT. It was not evident that the nanosheet morphology inhibited or delayed MMT phase separation to cristobalite and mullite.

The absence of (100) and (101) α‐quartz diffraction peaks in the SiOC‐1200 and SiOC–1400 samples further indicated that the α‐quartz phase was similarly MMT‐derived. Notably, the intensities of the (100) and (101) α‐quartz diffraction peaks in Figure 4 were similar among all MMT‐SiOC samples and therefore not dependent on the MMT content. However, α/β‐quartz are not known products from SiOC phase separation or MMT decomposition even in bulk/layered studies. Furthermore, as the tendency for MMT to decompose to cristobalite and mullite was not suppressed even as a nanosheet, it should not be expected for nanostructured MMT to yield α‐quartz at 900 °C. Neither originating from the nanomaterial reinforcement nor the ceramic matrix, the α‐quartz phase was determined to form at the interface of the amorphous SiOC matrix and the crystalline MMT nanosheet, likely during the polymer‐to‐ceramic transition. From the XRD spectra of MMT‐NH_2_ in Figure 2, the SiO_2_ monolayer that formed on the MMT nanosheet after AEAPTMS functionalization served as a potential surface for epitaxial SiO_2_ formation to bridge the interface between the crystalline nanosheet inclusion and the amorphous SiOC matrix. As the organic moieties were thermally cleaved from MMT‐NH_2_ between 200 and 700 °C, the exposed siliceous MMT nanosheet surface was able to react with the pyrolytically‐forming SiOC ceramic to form a homogenous microstructure. Without a proper interface, the MMT nanosheets would serve as inclusions rather than reinforcements and as a site for crack propagation and ceramic fracture/failure.

Above the α→β quartz inversion at 573 °C, the thermodynamically stable β‐quartz phase had a hexagonal crystal structure that maintained coherency with the hexagonal crystal structure of the MMT nanosheets. These events occurred simultaneously with the radical‐mediated polymer‐to‐ceramic transformation of the SiOC matrix and provided sites for interfacial bonding between exposed Si─O groups of MMT and the disordered Si tetrahedra of the evolving SiOC matrix. Furthermore, the high‐symmetry β‐quartz phase provided coherency to resolve the crystalline‐to‐amorphous microstructural transition at the interface. Further heating above 873 °C would induce a phase transformation from β‐quartz to tridymite depending on the presence of alkali/alkali earth metal impurities necessary to stabilize the tridymite phase, or to β‐cristobalite at 1470 °C through an intermediate amorphous phase.^[^ ^46^ ^]^ It was not apparent that the MMT‐SiOC interfacial regions transformed to β‐cristobalite or accelerated the decomposition of the MMT regions to cristobalite and mullite as evidenced by the absence of the 22° α‐cristobalite diffraction in the 1/2/5MMT‐1200 samples (Figure 4b). Therefore, the interface was postulated to exist as an amorphous silica boundary bridging the MMT nanosheets and the SiOC matrix. Cristobalite phases were derived from MMT phase decomposition and increased with MMT content. Cristobalite crystallization was observed in SiO_2_‐based ceramic cores at the surface of quartz grains at 1380 °C,^[^ ^47^ ^]^ and similarly proceeded in the 1/2/5MMT‐1400 ceramics at the quartz interfaces between the MMT and SiOC matrix. Of note, both β‐quartz and β‐cristobalite undergo inversions to lower symmetry forms at 573 °C and 250 °C upon cooling, respectively, and may induce ceramic fracture at significant concentrations. The α‐quartz phases identified in Figure 4 formed during the quartz inversion upon cooling – therefore, it should be noted that the evolved α‐quartz interphase may cause ceramic fracture due to significant volumetric reduction.

**Table** **1** summarizes the ceramic yield (C.Y.), volumetric shrinkage (V.S.), and density (ρ) of *x*MMT‐SiOC (*x* = 0, 1, 2, 5) samples pyrolyzed at 900 °C, 1200 °C, and 1400 °C in Ar. Ceramic yields of ≈77%, volumetric shrinkages of ≈39%, and densities of 1.58 g cm^−3^ were generally identical among SiOC‐900, 1MMT‐900, 2MMT‐900, and 5MMT‐900 samples and indiscernible within experimental standard deviation. SiOC‐900 and *x*MMT‐900 ceramics all exhibited ≈7% porosity after pyrolysis as determined with He pycnometry (see Table S1, Supporting Information), indicating that preceramic PSO used in this study yielded some residual porosity that was not affected by MMT content. Elevated pyrolytic temperatures of 1200 °C and 1400 °C yielded noticeable increases in ceramic yields and densities, and corresponding decreases in volumetric shrinkages, with the MMT content. SiOC‐1200 exhibited a C.Y. of 73.5% while 1MMT‐1200, 2MMT‐1200, and 5MMT‐1200 exhibited increased yields of ≈75.3%. The V.S. of SiOC‐1200, 1MMT‐1200, and 2MMT‐1200 were approximately ≈48%, but 5MMT‐1200 exhibited less shrinkage of 46% – this was manifested in the slight increase in density from 1.73 g cm^−3^ in SiOC‐1200 to 1.75 g cm^−3^ in 1/2/5MMT‐1200. Notably, the residual porosity decreased to ≈3% in all 1200 °C ceramics (see Table S1, Supporting Information) due to intrinsic densification mechanisms in SiOC. Similarly at 1400 °C, 1/2/5MMT‐1400 had slightly higher C.Y.s of ≈74.5% compared to 73.2% for SiOC‐1400. The decrease in V.S. from SiOC‐1400 (47.4%) to 1/2MMT‐1400 (≈46.6%) was nominal but starker in 5MMT‐1400 (45.6%). Densities slightly increased from 1.74 to 1.78 g cm^−3^ from SiOC‐1400 to 5MMT‐1400; however, all 1400 °C samples exhibited some inherent porosity after pyrolysis, which can be attributed to CO gas evolution from the SiOC amorphous matrix carbothermal reduction to β‐SiC above 1300 °C. However, differences in residual porosity among 1400 °C samples were not discernable within experimental error but remained between 3 and 4% (see Table S1, Supporting Information). As a result, there was a slight decrease in V.S. from 1200 to 1400 °C in all samples. While the pure SiOC matrix primarily exhibited mass loss between 900 and 1200 °C and remained relatively constant to 1400 °C, 1/2/5MMT‐SiOC showed continued mass loss until 1400 °C due to MMT phase decomposition and carbothermal synthesis of β‐SiC nanowhiskers.

**Table 1:** Ceramic yield (C.Y./%), volumetric shrinkage (V.S./%), and density (ρ/g cm^−3^) of xMMT‐SiOC nanocomposites pyrolyzed at 900, 1200, and 1400 °C in Ar. The error indicates the standard deviation of 4–5 replicates.

Ceramic Sample	900 °C/Ar			1200 °C/Ar			1400 °C/Ar		
	C.Y. [%]	V.S [%]	ρ [g cm^−3^]	C.Y. [%]	V.S [%]	ρ [g cm^−3^]	C.Y. [%]	V.S [%]	ρ [g cm^−3^]
SiOC	77.1 ± 0.8	39.9 ± 0.6	1.58 ± 0.02	73.5 ± 0.3	48.3 ± 0.7	1.73 ± 0.01	73.2 ± 0.6	47.4 ± 0.5	1.74 ± 0.01
1MMT	77.6 ± 0.8	40.0 ± 0.4	1.57 ± 0.02	75.5 ± 0.5	48.1 ± 0.7	1.75 ± 0.01	74.4 ± 0.5	46.7 ± 0.4	1.75 ± 0.02
2MMT	76.9 ± 0.9	39.2 ± 0.9	1.58 ± 0.02	75.3 ± 0.4	47.3 ± 0.7	1.75 ± 0.02	74.7 ± 0.4	46.6 + 0.5	1.76 ± 0.01
5MMT	77.7 ± 0.7	38.4 ± 0.7	1.58 ± 0.02	75.9 ± 0.3	46.3 ± 0.2	1.76 ± 0.01	74.4 ± 0.6	45.6 ± 0.5	1.78 ± 0.02

#### 2.2.2 Microstructural Analysis

**Figure 5:**
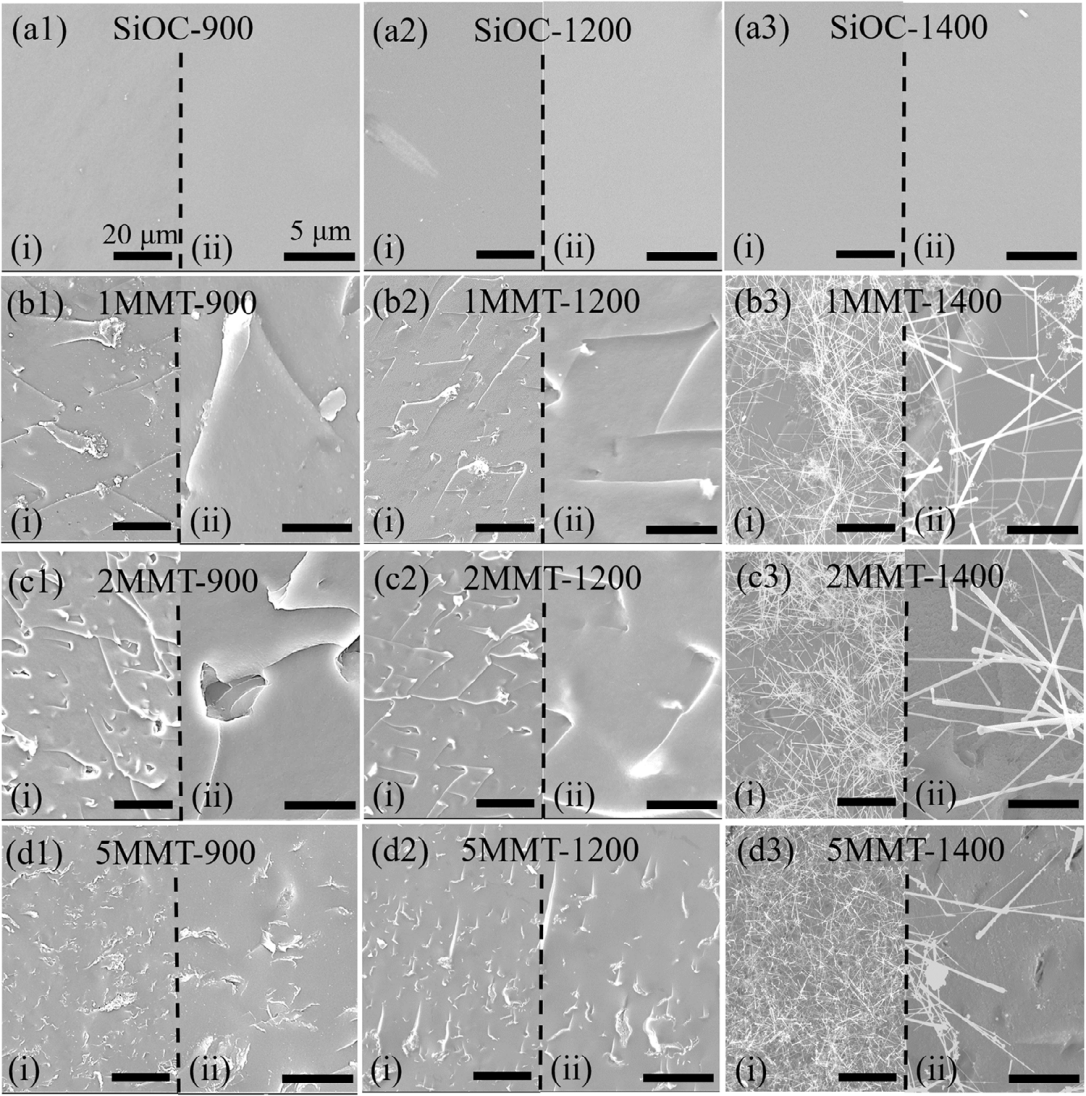
SEM micrographs of fracture surfaces of *x*MMT‐SiOC (*x* = 0, 1, 2, 5 wt.%) ceramics pyrolyzed to 900 °C, 1200 °C, and 1400 °C. Scale bars in (i) and (ii) in all panels are 20 and 5 µm, respectively.

**Figure 6:**
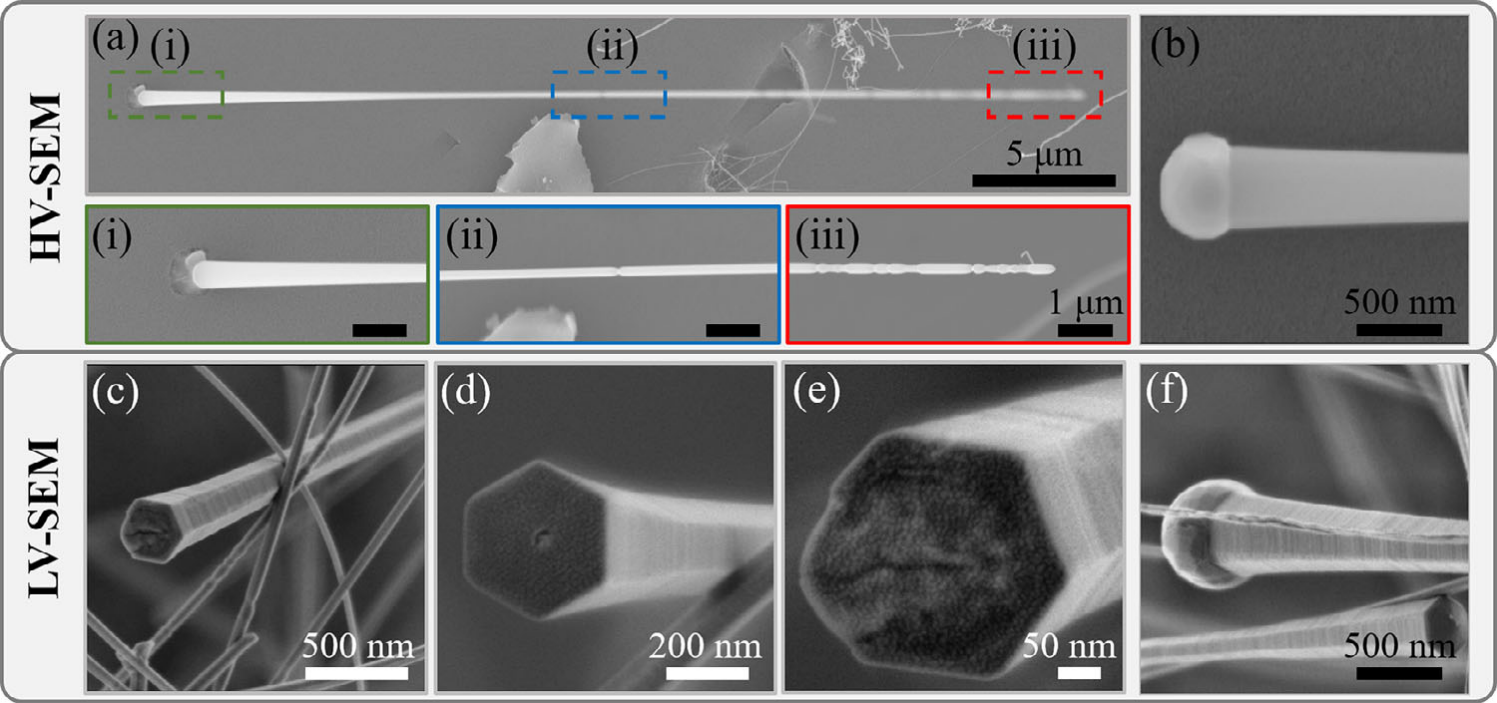
Morphology of β‐SiC nanowhiskers in 1/2/5MMT‐1400 ceramics observed with high voltage (10 kV – top) and low voltage (700 V – bottom) SEM. a) Unfractured nanowhisker grown from SiOC matrix with highlighted regions of (i) steady‐state, (ii) transition, and (iii) diffusion‐limited growth regimes. Scale bars in (i)‐(iii) are 1 µm. b) A pulled‐out nanowhisker from the SiOC matrix illustrating a nodule that served as a nanowhisker growth site. c–f) LV‐SEM imaging of SiC nanowhiskers revealing hexagonal morphology that was not resolvable with HV‐SEM.

Fracture surface morphologies investigated by SEM (**Figure** **5**) revealed that MMT contributed to increased faceting and the presence of crystallization fronts in 1/2/5MMT‐900 and 1/2/5MMT‐1200 samples. Compared to the smooth, featureless fracture surfaces of SiOC‐900 and SiOC‐1200, the crystallized facets indicated that the overall ceramic fracture mechanism was dominated by MMT‐derived α‐quartz domains rather than turbostratic C commonly present in PDC ceramics. Fracture surfaces of 1/2/5MMT‐1400 ceramics revealed an additional coating of nanowhiskers that were embedded in the ceramic matrix. The nanowhiskers were intertwined and protruded from the fracture surface. Additionally, 5MMT‐1400 ceramics exhibited slit‐like pores on the fracture surface from the MMT phase decomposition, which mirrored the fracture surface morphologies of MMT‐SiOC prepared with 30–40 wt.% bulk/calcined MMT as reported by Bernardo.^[^ ^28^ ^]^ Unfractured nanowhiskers (**Figure** **6**a) were able to reach at least 40 µm in length during pyrolysis at 1400 °C but varied in diameter, especially toward the tip. Nanowhisker growth appeared to proceed in (i) steady‐state and (iii) diffusion‐limited regimes with (ii) transition between the regimes after ≈20 µm of growth. Steady‐state growth was initiated at a nodule in the MMT‐SiOC matrix that was able to pull out from the matrix (Figure 6b) upon fracture. The nanowhiskers were typically 400–500 nm in diameter at the nodule and thinned to ≈200 nm toward the transition regime. Diameters varied between 100 and 200 nm in the diffusion‐limited growth regime, and the nanowhiskers were prone to fragmentation in this region. Nanowhisker growth appeared kinetically favorable at 1400 °C due to prevalence on 1/2/5MMT‐1400 fracture surfaces and continued non‐steady state growth after the transition zone, so it was postulated the nanowhiskers are SiC and the concentration of reactive C species for carbothermal synthesis of β‐SiC was insufficient for a high number of nanowhiskers present. This would further imply that the nanowhiskers are SiO_2_‐rich and originate from a SiO_2_ nodule. Low‐voltage SEM (LV‐SEM) imaging of the nanowhiskers (Figure 6c) uncovered hexagonal nanowhisker morphology that was not resolvable with usual SEM imaging (5–15 kV), further proving that it was SiC. The nanowhiskers consistently exhibited faceted growth, and exposed interfaces between the nodule and whisker (Figure 6d,e) showed a clear hexagonal growth surface. LV‐SEM of a pulled‐out SiC nanowhisker and nodule (Figure 6f) revealed hexagonal structural features that were obscured with HV‐SEM imaging (Figure 6b) from the conductive effects of SiC nanowhiskers.

**Figure 7:**
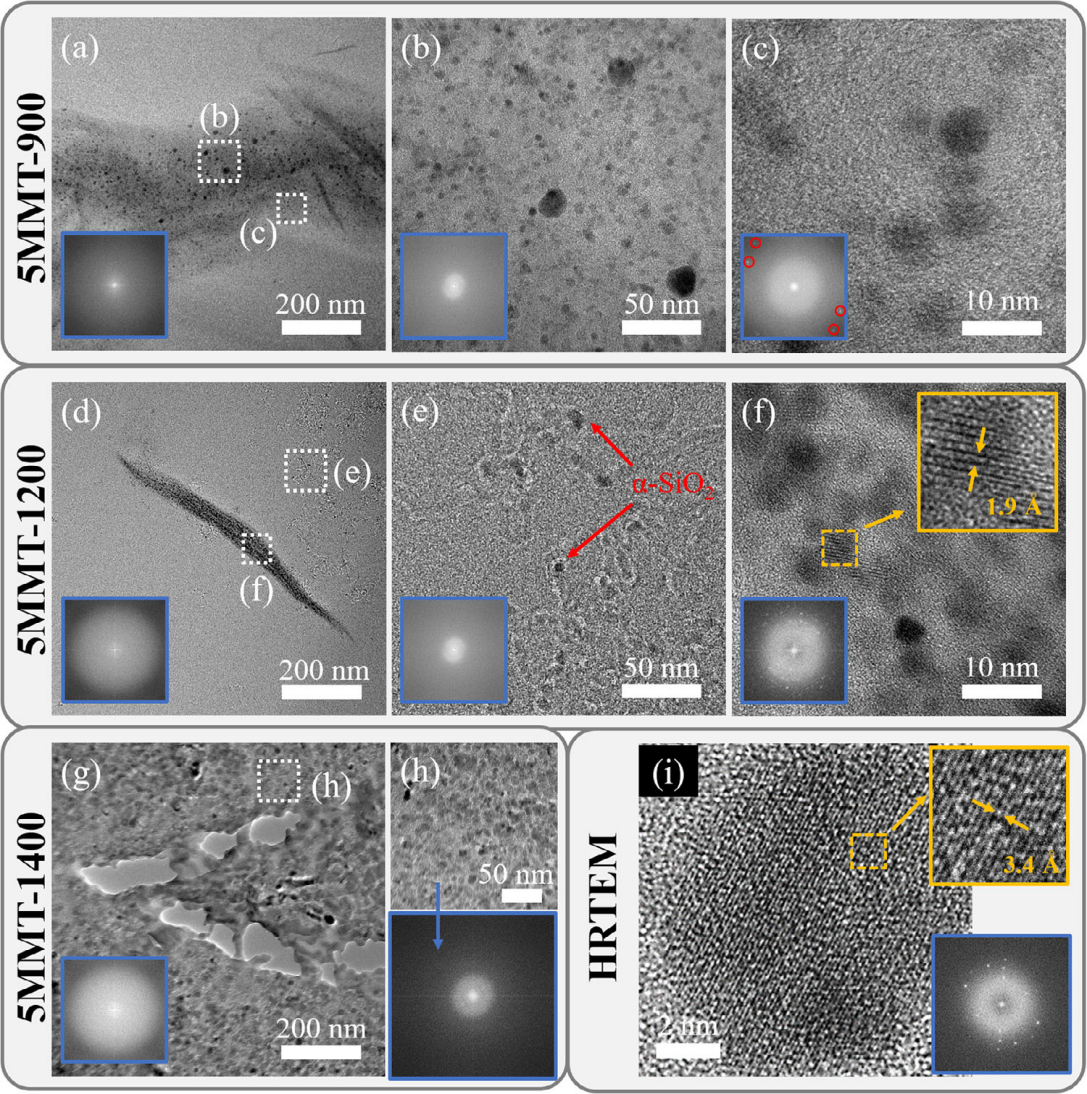
TEM micrographs illustrating nanostructural phase evolution of MMT nanosheets in 5MMT–900 a–c), 5MMT–1200 d–f), and 5MMT‐1400 g–h). Blue insets show corresponding FFT patterns and gold insets provide expanded/enhanced views of lattice fringes. HRTEM micrograph i) of typical α‐quartz nanoparticles observed in MMT domains. Scale bars: (a,d,g) 200 nm, (b,e,h) 50 nm, (c,f) 10 nm, and (i) 2 nm.

The nanostructural evolution of the MMT regions was analyzed with TEM (**Figure** **7**) in 5MMT‐900, 5MMT‐1200, and 5MMT‐1400, and revealed MMT nanoflakes randomly distributed in the SiOC matrix and α‐quartz nanoparticle evolution at the MMT‐SiOC interface. As anticipated, MMT nanosheet stacking observed in 5MMT‐900 (Figure 7a) and 5MMT‐1200 (Figure 7b) was attributed to the high MMT content in 5MMT‐900/1200/1400 but the random dispersion of the nanosheets pointed to the intrinsically exfoliated nature of the MMT nanosheets in MMT‐SiOC. As evidenced by XRD, α‐quartz nanoparticles were observed at the MMT‐SiOC interface and on the MMT nanoflakes in 5MMT‐900 (Figure 7b,c), which indicated that the SiO_2_ interlayer induced coherency between the MMT regions and the SiOC matrix. α‐quartz nanoparticles were generally ≈5 nm in size and randomly dispersed on the MMT nanosheet surface. As α‐quartz is a low temperature polymorph, nanoparticle size and distribution indicate crystallization from a high temperature polymorph such as the intermediate amorphous SiO_2_ phase upon cooling.

In 5MMT‐1200 (Figure 7f), the MMT nanosheets were covered with a higher concentration of α‐quartz nanoparticles than at the interface with the SiOC matrix. As supported by XRD (Figure 4b), pyrolysis to 1200 °C yielded a refined quartz phase; accordingly, the amorphous SiO_2_ interlayer experienced further refinement during pyrolysis to yield the higher concentration of α‐quartz nanoparticles. Kinetic analysis of quartz phase transformations revealed positive activation energies from quartz to intermediate amorphous SiO_2_ which would spur amorphous phase refinement with higher pyrolysis temperatures.^[^ ^46^ ^]^ The MMT nanosheets were more uniformly covered with α‐quartz nanoparticles, pointing to further refinement of the amorphous SiO_2_ interlayer. α‐quartz was characterized by lattice fringe spacing of 1.9 Å corresponding to the (112) plane (Figure 7f). HRTEM analysis (Figure 7i) further confirmed the presence of α‐quartz with (101) lattice spacings of 3.4 Å clearly observed. α‐quartz was also observed within the MMT‐SiOC interface (Figure 7e) and connected by amorphous SiO_2_ veins. We believe that both amorphous and α‐quartz formed in this region to maintain a coherent interface between MMT domains and SiOC, potentially through retained free C and new SiOC phases.

MMT phase decomposition and SiOC conversion to SiC at 1400 °C were observed with slit‐like pore formation from the MMT nanosheets and SiO_2_ nucleation in the SiOC matrix (Figure 7g). Pore sizes obtained from TEM were not truly representative due to pore smearing during FIB thinning but regardless illustrated that the microstructural degradation in MMT‐SiOC was primarily driven by MMT phase separation. The SiOC matrix surrounding the MMT‐derived pores (Figure 7h) exhibited amorphous SiO_2_ nodules, but was also expected from the observed amorphous SiO_2_ halo in Figure 4c in pure SiOC. Overall, 1400 °C pyrolysis was primarily characterized by MMT decomposition and slit‐like pore evolution in xMMT‐1400 ceramics. Amorphous SiO_2_ refinement at the MMT‐SiOC interface predominantly occurred between 900 and 1200 °C, which yielded the increased α‐quartz content observed in the XRD spectra of the MMT‐SiOC ceramics. However, amorphous SiO_2_ inherently present in SiOC did not undergo a similar phase transformation upon cooling, so the observed crystallization was assumed to be induced by the hexagonally‐structured MMT surface serving as nucleation sites. In addition, at the expense of SiO_2_ refinement, the interface was presumably carbon‐deficient and may have less SiOC tetrahedra.

### 2.3 Etching‐Induced Porous SiOC/SiC Ceramics

Porous SiOC/SiC ceramics were prepared by etching of SiO_2_ domains with excess HF (Equation 3), and the effect of MMT content on selectively‐etched porosity and specific surface area (SSA) in *x*MMT‐SiOC ceramics was determined with N_2_ sorption experiments (Figure 7). As Si─C bonds are resistant to HF, selectively etched porosity was generally representative of the size and distribution of SiO_2_ domains present in the MMT‐SiOC ceramics.

(3) $$\mathrm{Si}O_{2}+6\mathrm{HF}\to H_{2}\mathrm{Si}F_{6}+2H_{2}O$$

**Figure 8:**
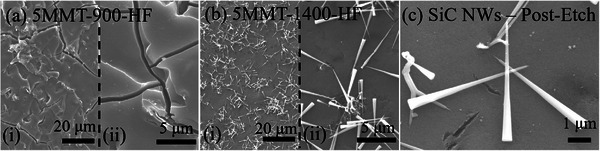
SEM micrographs of HF‐etched a) 5MMT‐900 and b) 5MMT‐1400 ceramic samples. c) SiC nanowhiskers on the surface of 5MMT‐1400 after HF etching.

**Figure 9:**
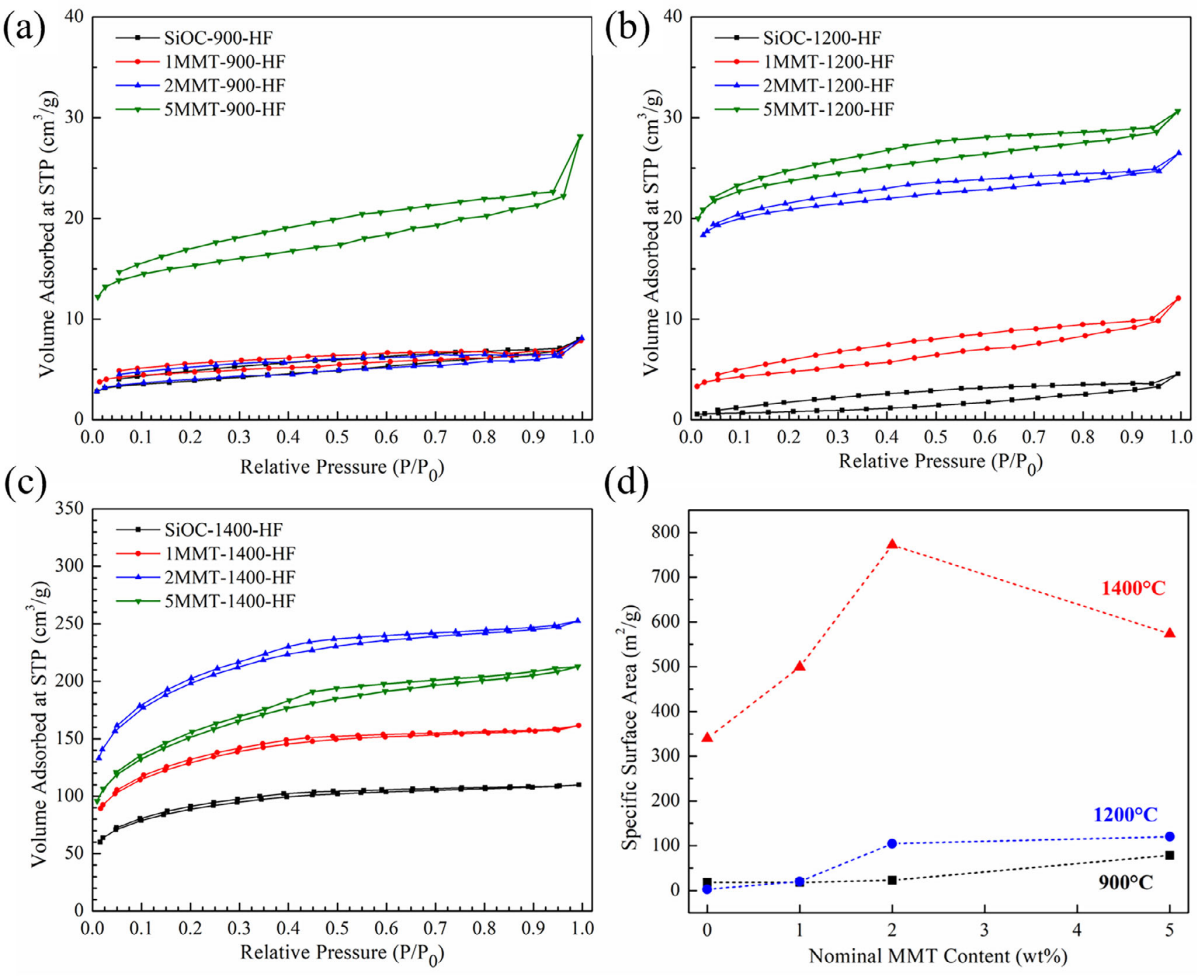
Nitrogen sorption isotherms of HF‐etched *x*MMT‐SiOC ceramics (*x* = 0/black, 1/red, 2/blue, 5/green) pyrolyzed at a) 900 °C, b) 1200 °C, and c) 1400 °C. d) SSAs of HF‐etched *x*MMT‐SiOC ceramics pyrolyzed at 900 °C (black), 1200 °C (blue), and 1400 °C (red) as determined by NLDFT fitting.

SiOC‐900‐HF, 1MMT‐900‐HF, and 2MMT‐900‐HF ceramic samples exhibited very little porosity with nearly identical gas adsorption volumes of 8.0, 7.9, and 8.1 cm^3^ g^−1^, indicating that the evolved SiO_2_ domains from MMT were quite minimal and indiscernible from those present in SiOC‐900. While 5MMT‐900‐HF exhibited increased gas sorption (28.1 cm^3^ g^−1^), the HF‐etched samples were quite brittle. Microstructural analysis (**Figure** **8**a) revealed microcracks present after etching. The minimal porosity observed in SiOC‐900‐HF was attributed to the small α‐quartz phase present from residual –OH groups, which were generally invariant with 1% and 2% MMT content. At 5% MMT content, microcracking may be due to residual stresses from coalesced α‐quartz domains and gas release during etching. Specific surface areas (**Figure** **9**d) of SiOC‐900‐HF, 1MMT‐900‐HF, and 2MMT‐900‐HF of 18, 18, and 23 m^2^ g^−1^, respectively, were indicative of poorly refined SiO_2_ phases. The SSA of 5MMT‐900‐HF of 79 m^2^ g^−1^ was reflective of microcracking and aggregated SiO_2_ regions. The calculated porosities (Table S2, Supporting Information) of ≈8‐10% did not appreciably change after HF etching, indicating that while a small amount of SiO_2_ was removed during etching, porosity at 900 °C was predominantly a function of the residual porosity after pyrolysis.

Pyrolysis at 1200 °C led to a refinement of α‐quartz domains, and the 1/2/5MMT‐1200‐HF ceramics displayed improved pore characteristics compared to SiOC‐1 (Figure 9b). While SiO_2_ clusters are present in SiOC ceramics when measured by ^29^Si NMR,^[^ ^48^, ^49^ ^]^ these nanosized clusters (<2 nm) were too small or inaccessible to be etched by HF. The small concentration of SiO_2_ in SiOC‐1200 was reflected in poor gas sorption volume (4.6 cm^3^ g^−1^) and SSA (3 m^2^ g^−1^) for SiOC‐1200‐HF. Improved porosities in 1/2/5MMT‐1200‐HF ceramics resulted from a refinement of MMT‐derived α‐quartz domains, with gas sorption volumes and SSAs increasing to 12.1, 26.5, and 30.6 cm^3^ g^−1^ and SSA 20, 105, and 120 m^2^ g^−1^, respectively. 1/2/5MMT‐1200‐HF ceramics exhibited ≈5‐6% porosity compared to 3% in SiOC‐1200‐HF (Table S2, Supporting Information). The effect of MMT on the porosity plateaued between 2% and 5% MMT content due to either limited diffusional lengths by interspersed turbostratic C or tactoid formation of MMT layers from increased MMT loading.

Pore characteristics increased by an order of magnitude between 1200 and 1400 °C inherently due to SiO_2_ phase refinement from carbothermal reduction as shown by the amorphous SiO_2_ halo emergence in Figure 4c and improved gas sorption volume of 109.9 cm^3^ g^−1^ and SSA of 340 m^2^ g^−1^ for SiOC‐1400‐HF (Figure 9c). While the MMT content improved selectively etched porosity in 1/2MMT‐1400‐HF with increased sorption volumes of 161.7 cm^3^ g^−1^ and 252.5 m^2^ g^−1^, 5MMT‐1400‐HF displayed a reduced sorption volume of 212.7 cm^3^ g^−1^. The SSAs of 1/2/5MMT‐1400‐HF ceramics followed the same trend, increasing to 500 m^2^ g^−1^ and 772 m^2^ g^−1^ before decreasing to 574 m^2^ g^−1^, respectively. The decrease in pore characteristics may be attributed to the inherent macroporosity visible on the fracture surface of 5MMT‐1400 (Figure 5) due to tactoidal layering of MMT nanoflakes at 5 wt.% loading and MMT decomposition above 1350 °C. The calculated porosities increased similarly with MMT content, from 5% in SiOC‐1400‐HF up to 17% in 2MMT‐1400‐HF and 23% in 5MMT‐1400‐HF (Table S2, Supporting Information). The observed increase in pore characteristics in 1/2/5MMT‐1400‐HF was a combination of inherent porosity from MMT decomposition in the unetched 1/2/5MMT‐1400 ceramics (Figure 3) and etching of SiOC‐derived and MMT‐derived SiO_2_ regions.

**Figure 10:**
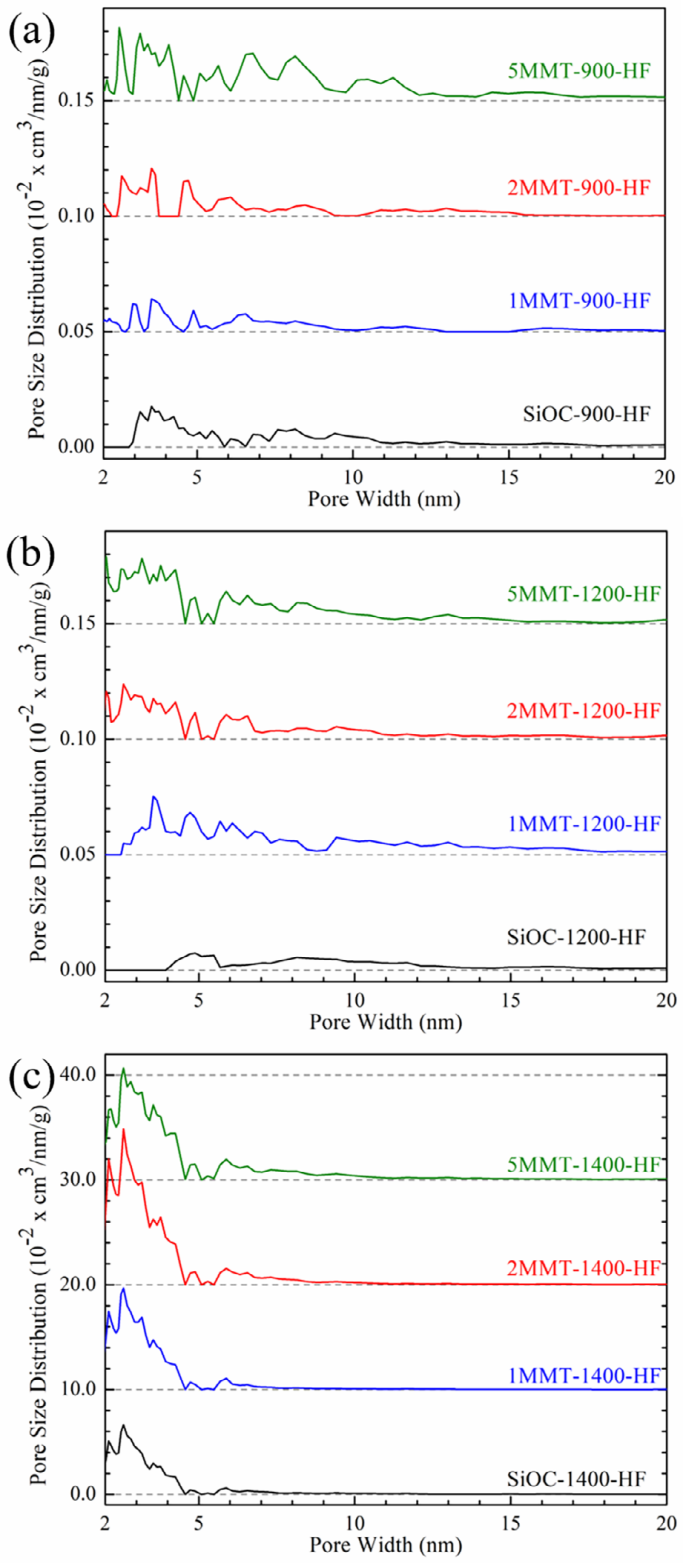
Pore size distributions of HF‐etched MMT‐SiOC ceramics pyrolyzed at a) 900 °C, b) 1200 °C, and c) 1400 °C. Curves were offset for clarity.

Pore size distributions (**Figure** **10**) were determined from a NLDFT model of the adsorption branch of N_2_ isotherms assuming cylindrical and sphere pore structures. The minimal pore characteristics of *x*MMT‐900‐HF ceramics (Figure 10a) were primarily due to the underlying porosity from unresolved Si─OH bonds in the SiOC matrix, with the small concentration of pores in SiOC‐900‐HF sized between 3 and 10 nm. The addition of MMT generally increased the pore sizes between 2 and 5 nm although quite minimally at 900 °C. Further pyrolysis at 1200 °C coincided with a refinement of SiO_2_ clusters (Figure 10b), yielding few ≈5 nm pores upon pyrolysis that were representative of the SiO_2_ phases inherently present in SiOC‐1200. MMT content primarily increased pore concentrations between 2 and 5 nm in 1/2/5MMT‐1200‐HF with slight additional contributions to pore sizes from 6 to 10 nm, indicating that the selectively‐etched porosity was primarily due to the inclusion of MMT nanosheets and independent of matrix effects.

Pore size distributions in *x*MMT‐1400‐HF ceramics (Figure 10c) showed that the porosity from MMT‐derived SiO_2_ domains augmented that intrinsic to SiOC‐1400‐HF ceramics. Carbothermal reduction above 1300 °C decreases SiO_2_ domains to 2 to 5 nm, which were shown to solely contribute to the pore size characteristics of SiOC‐1400‐HF. Pore size concentrations between 2 and 5 nm further increased in 1/2MMT‐1400‐HF from the additive effect of MMT‐derived SiO_2_ domains. Correlating with the decrease in gas sorption volume and SSA, pore size concentrations in 5MMT‐1400‐HF were generally similar to those in 1MMT‐1400‐HF and attributed to some loss of the nanostructured MMT‐derived SiO_2_ domains in the development of slit‐like pores observed under SEM (Figure 5). While the microstructures of etched *x*MMT‐SiOC ceramics were generally invariant of their pre‐etched analogs, the morphologies of β‐SiC nanowhiskers in all *x*MMT‐1400‐HF ceramics were affected by HF etching with the nodule present at the base of the nanowhiskers disappearing and the overall length refining to ≈5 µm (Figure 8b,c). The sharpened β‐SiC nanowhiskers upon etching indicated that the original nanowhiskers originated from a SiO_2_ nodule. The SiO_2_‐rich nature of the nanowhiskers was postulated to also contribute to non‐steady state growth regimes observed as the lack of available reactive C species present during pyrolysis at 1400 °C precluded continuous carbothermal reduction of SiO_2_ for β‐SiC crystallization.

*x*MMT‐1200‐HF ceramics displayed porosity indicative solely of MMT‐derived SiO_2_ domains, while the porosity in *x*MMT‐1400‐HF ceramics was developed from MMT phase decomposition and etching of intrinsic amorphous SiO_2_ phase and residual MMT‐derived SiO_2_ domains. SSAs of *x*MMT‐1200‐HF ceramics remained below 100 m^2^ g^−1^, so MMT‐derived SiO_2_ phase development may be diffusion‐limited by turbostratic C barriers in the SiOC matrix. Reactive fillers or pyrolysis atmospheres may be required to augment or improve pore volumes. Overall, 1MMT‐1200‐HF and 2MMT‐1200‐HF ceramics serve as potential candidates in porous ceramic applications due to the controlled porosity achieved from the dispersion and etching of the MMT nanosheets.

## 3 Conclusions

Organophilic MMT nanoflakes were synthesized by novel twice‐functionalization of MMT nanosheets in order to promote exfoliation in the preceramic polymer matrix. Novel MMT‐SiOC ceramic nanocomposites were prepared to investigate the effect of nanostructured MMT on phase evolution and selectively etched porosity between 900 and 1400 °C. *x*MMT‐SiOC ceramic nanocomposites fabricated at 900 °C and 1200 °C showed growth and refinement of a crystalline α‐quartz phase on the MMT nanoflakes that led to the formation of 2 to 5 nm pores upon etching. SSAs of HF‐etched *x*MMT‐1200 ceramics reached ≈100 m^2^ g^−1^ but the size of the SiO_2_ domains appeared to be diffusion‐limited by turbostratic C phases in the SiOC matrix. Further pyrolysis to 1400 °C led to β‐SiC nanowhisker formation in *x*MMT‐1400 ceramics with structures observed up to 20 µm in length and 500 nm in diameter. Low voltage SEM imaging revealed hexagonal faceting during nanowhisker growth. The findings of this study offer novel fabrication of exfoliated MMT nanocomposites and show that nanostructured MMT and MMT‐SiOC ceramics are attractive candidates for porous ceramics and precursors to nanostructured SiC.

## 4 Experimental Section

### Materials

Naturally occurring montmorillonite clay (Crude MMT, 200 mesh powder, Alfa Aesar, Ward Hill, MA) was chosen as the crude clay precursor. Sodium hydroxide (NaOH, 50 wt.% in H_2_O, Sigma‐Aldrich, Burlington, MA), acetic acid (CH_3_COOH, glacial, Fisher Chemical, Waltham, MA), and silver nitrate (AgNO_3_, ReagentPlus, ≥99.0%, Sigma‐Aldrich, Burlington, MA) were used in washing and titrant solutions. Cetyltrimethylammonium bromide (CTAB, ≥98%, Sigma‐Aldrich, Burlington, MA) and AEAPTMS (AEAPTMS, 98%, Gelest, Inc., Morrisville, PA) were chosen to surface functionalize the exfoliated MMT nanoflakes. A commercial polysiloxane Polyramic SPR‐684 (PSO, [‐Si(Ph)_2_O‐]_3_[‐Si(Me)(H)O‐]_2_[‐Si(Me)(CH = CH_2_)O‐]_2_, Starfire Systems, Inc., Glenville, NY) was chosen as the base preceramic polymer, with a 2.1% platinum‐divinyltetramethyldisiloxane complex in xylene (Karstedt's catalyst, Gelest, Inc., Morrisville, PA) selected to crosslink the preceramic green bodies. Concentrated hydrofluoric acid (HF, 49 wt.%, Avantor Specialty Materials, Inc., Radnor, PA) was used to selectively etch SiO_2_ from the MMT‐SiOC ceramics. Acetone (technical grade, Fisher Scientific, Waltham, MA), dimethyl sulfoxide (DMSO, ≥99.9%, Sigma‐Aldrich, Burlington, MA), hexanes (anhydrous, 95%, Sigma‐Aldrich, Burlington, MA), methanol (MeOH, 200 proof, Fisher Scientific, Waltham, MA), toluene (≥99.5%, Fisher Scientific, Waltham, MA), and ultrapure water (NP H_2_O, 18.2 MΩ‐cm) were used throughout the experiment as general solvents. Argon (Ar, Industrial Grade, Airgas, Christiansburg, VA) was used for the pyrolysis atmosphere. All chemicals were used without further purification.

### MMT Purification and Exfoliation

Exfoliated MMT nanosheets were collected through a modified procedure inspired by prior literature.^[^ ^20^, ^21^ ^]^ A 5 w/v% suspension of crude MMT in 1.0 M NaOH_(aq)_ was mechanically stirred for 12 h, after which the alkalized MMT powders were washed with 0.05 M CH_3_COOH_(aq)_ until the pH of the effluent was between 6–7. The purified MMT powder was collected over a 0.45 µm hydrophobic PVDF filter with 70% EtOH_(aq)_ and vacuum‐dried (Fisher Isotemp Model 281A Vacuum Oven, Waltham, MA) at 70 °C for 12 h. To exfoliate MMT nanosheets, a 0.5 w/v% suspension of purified MMT in NP H_2_O was frozen at −20 °C. The suspensions were thawed at room temperature and immersion bath‐sonicated (Cole‐Parmer Model 08895‐01, 50 W, Vernon Hills, IL) for 1 h; five cycles of freezing‐thawing‐sonication (F/T/S) were performed. The exfoliated MMT nanosheets were collected after centrifugation (Sorvall Legend X1R with FiberLite 8 × 50c rotor, Thermo Fisher Scientific, Waltham, MA) at 11 000 *x* g for 15 min. The exfoliated MMT nanosheets (MMT‐Na^+^) were collected after lyophilization (FreeZone Plus 2.5 L, Labconco Corp., Kansas City, MO) for 48–72 h and stored under vacuum.

### Exfoliated MMT Functionalization

Exfoliated MMT nanopowders were further surface functionalized with CTAB and AEAPTMS to improve dispersion in the preceramic polymer through modification and synthesis of prior reported procedures.^[^ ^30^, ^36^, ^50^ ^]^ To a 0.5 w/v% suspension of MMT‐Na^+^ in NP H_2_O lightly stirring (<200 rpm) at 50 °C, a 20 w/v% CTAB_(MeOH)_ was charged and stirred at 50 °C for 3 h. The CTAB_(MeOH)_ solution corresponded to a 2X mass excess of CTAB to MMT‐Na^+^. The CTAB‐exchanged MMT nanosheets (MMT‐CTA^+^) were washed repeatedly with MeOH until no halide ions were detected with 0.1 M AgNO_3(aq)_, collected over a 0.45 µm hydrophilic PVDF filter with NP H_2_O, and dried under vacuum at 100 °C for 12 h.

MMT‐CTA^+^ nanosheets were further functionalized with AEAPTMS to form a covalently bonded hydrophobic surface on the MMT nanosheets. A 0.5 w/v% suspension of MMT‐CTA^+^ in 90% DMSO‐10% MeOH was stirred in a 30 °C water bath, and glacial CH_3_COOH was slowly added to obtain a pH of 4. A 10 w/v% AEAPTMS_(EtOH)_ solution – corresponding to a 2X mass excess with respect to MMT‐CTA^+^ – was added dropwise alternatively with additional glacial CH_3_COOH to maintain a pH of 4, and stirred for 3 h at 30 °C. The twice‐functionalized silane‐functionalized MMT nanoflakes (MMT‐NH_2_) were washed 5 times with 50% acetone‐50% MeOH, collected over a 0.45 µm hydrophobic PVDF filter with hexanes, and vacuum‐dried at 80 °C for 12 h. It should be noted that crude MMT refers to bulk/layered MMT that is comprised of many layers, while MMT‐NH_2_ refers to only the single‐to‐few flake thick MMT nanosheets obtained after F/T/S exfoliation and centrifugation.

### MMT‐SiOC Sample Preparation

*x*MMT‐PSO preceramic green bodies (*x* = 0, 1, 2, 5 wt.%) were prepared by mixing PSO with toluene (≈50 v/v% relative to PSO), 100 ppm of Karstedt's catalyst (relative to PSO), and the MMT‐NH_2_ nanoflakes. The suspension was stirred for at least 8 h under vacuum to remove residual solvent. The polymer suspensions were cast to Φ12 mm x 6 mm cylindrical silicone molds, degassed under vacuum, and cured at 60 °C for 6 h and 120 °C for 12 h. The green bodies were lightly ground and polished up to 1500 grit with SiC abrasive pads to remove any mold artifacts, and the edges were slightly beveled. To minimize potential surface oxidation, the green bodies were placed between two sheets of carbon felt in an alumina crucible. Under a flowing Ar environment (≈500 mL min^−1^), the green bodies were pyrolyzed at 900 °C, 1200 °C, or 1400 °C in a horizontal tube furnace (1830‐12 Horizontal Tube Furnace, CM Furnaces, Inc., Bloomfield, NJ), with heating and cooling rates of 2 °C min^−1^ and soaking time of 2 h. The MMT‐SiOC ceramic samples were denoted as *x*MMT‐T_Pyr_ with *x* = 1, 2, 5 wt.% and T_Pyr_ = 900, 1200, or 1400 °C. The blank SiOC samples without MMT were similarly denoted SiOC‐T_Pyr_.

### Selective Etching in MMT‐SiOC Ceramics

Pyrolyzed *x*MMT‐T_Pyr_ samples were lightly fractured into ≈5 mm pieces and etched in a stirring 10% HF (aq) solution at less than 5 wt.% for at least 96 h, or until no mass loss was observed. Samples were thoroughly washed with NP H_2_O and dried under vacuum at 120 °C for 12 h.

### Characterization

Ceramic yield and volume shrinkage were calculated from changes in mass and sample volume, respectively, between green bodies and pyrolyzed samples. Reported values are averages of 4–6 replicates and corresponding errors indicate standard deviations. The densities of pyrolyzed *x*MMT‐SiOC samples were measured using the Archimedes principle with EtOH as the immersion medium. Skeletal densities were obtained in replicate (n≥5) with helium pycnometry (AccuPyc II 1340, Micromeritics Instrument Corp., Norcross, GA, USA) with a 1‐cc sample cell and an outlet pressure of 134.4 kPa (see Supporting Information). The phase compositions of powdered samples were analyzed with an X‐ray diffractometer (XRD, Empyrean, Malvern Panalytical Ltd., Almelo, The Netherlands) operated at 40 kV and 40 mA with Cu Kα radiation between 2θ = 2–90° and a scan rate of 0.02° s^−1^. Microstructures were observed with a scanning electron microscope at 10 kV (SEM, IT‐500HR, JEOL USA, Peabody, MA, USA) and a transmission electron microscope (TEM, JEOL 2100, JEOL USA, Peabody, MA, USA) equipped with a LaB_6_ filament operated at 200 kV. SiC nanostructure morphologies were observed with low voltage (700 V) imaging (LV‐SEM, GeminiSEM 460, Carl Zeiss AG, Oberkochen, Germany). TEM lamella of 5MMT‐SiOC ceramics were prepared by focused‐ion beam liftout and thinning (FIB, Helios 5 UC, Thermo Fisher Scientific, Inc, Waltham, MA, USA). Thermogravimetric analysis (TGA, TGA 5500, TA Instruments, New Castle, DE, USA) of exfoliated/functionalized MMT nanoflakes was performed with a heating rate of 5 °C min^−1^ from 50 to 1000 °C under argon flow of 40 mL min^−1^. Bond characteristics in the functionalized MMT nanoflakes were analyzed by Fourier‐transformed infrared spectroscopy (FTIR, Varian 670‐IR, Agilent Technologies, Inc., Santa Clara, CA, USA) with the attenuated total reflectance (ATR) method between 4000 and 600 cm^−1^ with a resolution of 4 cm^−1^ and averaged over 128 scans. Pyrolytic mass loss and heat flow of *x*MMT‐PSO green bodies were analyzed by a simultaneous thermal analyzer (SDT, SDT 650, TA Instruments, New Castle, DE, USA). Porosity and surface area characteristics were determined by nitrogen adsorption at 77 K (Quantachrome Autosorb‐iQ C‐XR, Quantachrome Instruments, Boynton Beach, FL, USA). Samples were degassed under vacuum at 250 °C for 12 h prior to analysis. A Non‐Local Density Functional Theory (NLDFT) analytical model was applied to the adsorption branch with a spherical/cylindrical pore structure based on a silica adsorbate model to determine pore characteristics. Young's modulus and hardness were determined by nanoindentation (Nano Indenter G200, Keysight Technologies Inc., Santa Rosa, CA, USA) with a Berkovich diamond indenter to a peak displacement of 1 µm and averaged across a 5 × 5 array with 20 µm indent spacing (see Supporting Information). Samples were mounted in epoxy and polished up to 0.1 µm with diamond suspension prior to analysis.

## Conflict of Interest

The authors declare no conflict of interest.

## Author Contributions

A.V.R.: conceptualization, methodology, validation, formal analysis, investigation, resources, data curation, writing – original draft, writing – review & editing, and visualization. K.L.: conceptualization, resources, writing – review & editing, supervision, project administration, and funding acquisition.

## Supporting information



Supporting Information

## Data Availability

The data that support the findings of this study are available from the corresponding author upon reasonable request.
