# Supplementary material for: Twice‐Functionalized Montmorillonite Nanosheets for Polymer‐Derived MMT‐SiOC Nanocomposites: Phase Formation and Porosity
Source: Small. 2025 Apr 7;21(19):2408218. doi: 10.1002/smll.202408218 (PMC12067154; doi:10.1002/smll.202408218)
Supplement: Supplementary file 1 — Supporting Information [file SMLL-21-2408218-s001.docx]

**Supplementary Information**

**Twice-Functionalized Montmorillonite Nanosheets for Polymer-Derived MMT-SiOC Nanocomposites: Phase Formation and Porosity**

Advaith V. Rau^a^, Kathy Lu^a,b^*

^a^Department of Materials Science and Engineering, Virginia Polytechnic Institute and State University, Blacksburg, Virginia, 24061, USA

^b^Department of Mechanical and Materials Engineering, University of Alabama-Birmingham, Birmingham, AL, 35294, USA

*Corresponding author; Email: klu@uab.edu

# S-1. Porosity of MMT-SiOC and MMT-SiOC-HF Ceramics

Table S-1: Bulk and skeletal densities (g·cm^-3^) and calculated porosities (%) of *x*MMT-SiOC ceramics pyrolyzed from 900°C to 1400°C. Errors for bulk and skeletal densities represent standard deviations of replicate measurements, and error in porosity was determined by error propagation.

Table S-2: Bulk and skeletal densities (g·cm^-3^) and calculated porosities (%) of *x*MMT-SiOC ceramics after HF etching. Errors for bulk and skeletal densities represent standard deviations of replicate measurements, and error in porosity was determined by error propagation. Values for 5MMT-900-HF were not obtainable as the sample was too brittle and broke during measurements.

# S-2. Nanoindentation of MMT-SiOC Ceramics

Nanoindentation revealed that both MMT content and pyrolysis temperature affected the mechanical properties of MMT-SiOC ceramics, specifically relating to MMT-SiOC interface. At 900°C, increasing MMT content at 1 and 5 wt% induced reductions in both elastic modulus (E) (1MMT-900: 57±3 GPa; 5MMT-900: 53±1 GPa) and hardness (H) (1MMT-900: 6.6±0.7 GPa; 5MMT-900: 6.4±0.2 GPa) compared to SiOC-900 (E: 58±1 GPa, H: 7.1±0.2 GPa). However, at 1200°C, MMT content yielded net improvements to elastic moduli and hardness in 1MMT-1200 (E: 78±3 GPa, H: 8.5±0.5 GPa) and 5MMT-1200 (E: 77±5 GPa, H: 8.6±0.7 GPa) compared to SiOC-1200 (E: 71±4 GPa, H: 8.0±0.7 GPa). The general improvements in elastic modulus and hardness between SiOC-900 and SiOC-1200 was primarily attributed to the improved densification and reduction in porosity observed between 900°C and 1200°C (Table S-1) and postulated relaxation of the glassy SiOC matrix [1], but the seemingly dual nature of MMT in regards of mechanical properties pointed to the states and roles of the MMT-SiOC interface and the segregated C phase as the primary strengthening (or weakening) mechanisms.

The improved mechanical properties of SiOC glasses over analogous vitreous SiO_2_ are derived from incorporation of sp^3^-hybridized C into the SiO_2_ network that improves the connectivity of the glassy SiOC phase [1]. Segregated C content (i.e., sp^2^-hybridized turbostratic C) however has a mitigating effect on mechanical properties as ceramic deformation proceeds through shearing of turbostratic C layers. Between 900°C and 1200°C, the free C phase undergoes some refinement from isolated polycyclic aromatic C clusters to turbostratic C regimes [1]. However, free C phase refinement does not explain the trend observed in 1200°C ceramic samples. The trends in modulus and hardness were thus attributed to improved interfacial bonding between MMT and SiOC and refinement of interfacial α-quartz. At 900°C, unresolved Si-OH bonds limited the extent of interfacial bonding between MMT domains and SiOC, and the increased α-quartz content with MMT loading generally weakened the MMT-SiOC ceramics. However, further pyrolysis to 1200°C corresponding with H_2_ evolution led to increased interfacial α-quartz density as observed in TEM (Figure 7(f)) that ultimately strengthened the MMT-SiOC interface. While interfacial α-quartz may limit the strengthening effect of MMT at 1200°C, refinement of the MMT-SiOC interface ultimately created more bonding sites between MMT domains and SiOC to provide a slight yet discernable improvement to both E and H values.

1400°C pyrolysis led to a general decrease in E and H values from 1200°C pyrolyzed ceramics, primarily due to SiOC phase separation [1], pore evolution and MMT decomposition as elastic moduli and hardness values of SiOC-1400 (E: 56±4 GPa; H: 6.0±0.7 GPa), 1MMT-1400 (E: 58±5 GPa; H: 6.7±0.7 GPa), and 5MMT-1400 (E: 52±5 GPa; H: 6.2±0.8 GPa) were comparable to 900°C analogs. While in pure SiOC the decrease may be attributed to CO gas evolution and increasing segregated C content, 1MMT-1400 and 5MMT-1400 ceramics were primarily weakened by MMT decomposition, most evidently seen with the slit-like pores in 5MMT-1400 (Figure 5). Overall, 1200°C pyrolysis was required to obtain the best mechanical improvements due to improved interfacial bonding of MMT domains and the SiOC matrix through refinement of interfacial α-quartz.

Table S-3: Young’s moduli (E/GPa) and hardness (H/GPa) values for SiOC, 1MMT, and 5MMT ceramics pyrolyzed from 900°C to 1400°C. Values represent mean and standard deviation of n=20-25 indents. Indents with E and H values with a COV>2.5 were considered outliers and disregarded.

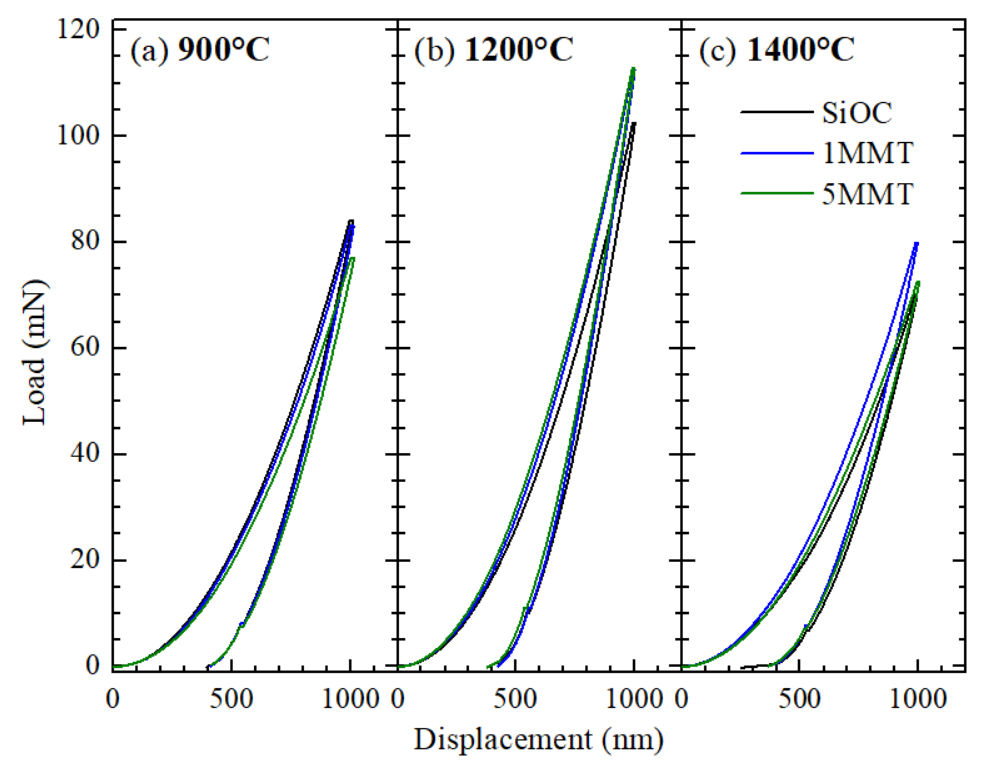


Figure S-1: Representative load-displacement curves from nanoindentation of SiOC (black), 1MMT (blue), and 5MMT (green) ceramics pyrolyzed at (a) 900°C, (b) 1200°C, and (c) 1400°C.

**References**

1. Stabler, C., et al., *Silicon oxycarbide glasses and glass-ceramics: “All-Rounder” materials for advanced structural and functional applications.* Journal of the American Ceramic Society, 2018. **101**(11): p. 4817-4856.
